# Community-based delivery of lifesaving maternal and newborn health interventions in vulnerable Ethiopian communities: Lessons from implementation research

**DOI:** 10.1371/journal.pone.0360788

**Published:** 2026-11-05

**Authors:** Gizachew Tadele Tiruneh, Agumasie Semahegn, Alemnesh Hailemariam Mirkuzie, Nebreed Fesseha, Addis Girma, Mikiyas Teferi, Hillina Tadesse, Shegaw Mulu, Biruhtesfa Bekele, Abdulhalik Workicho, Mirkuzie Woldie, Temesgen Ayehu, Biruk Bogale, Bezawit Mesfin Hunegnaw, Chala Tesfaye, Zemzem Mohammed, Mariamawit Asfaw, Lidiya Tefera, Frank DelPizzo, Addis Tamire, Misrak Mekonnen, Mesele Damte Argaw, Dessalew Emaway

**Affiliations:** 1 JSI Research & Training Institute, Inc., Addis Ababa, Ethiopia; 2 Amref Health Africa in Ethiopia, Addis Ababa, Ethiopia; 3 Fenot: Harvard T. H. Chan School of Public Health, Addis Ababa, Ethiopia; 4 Minstry of Health, Addis Ababa, Ethiopia; 5 Gates Foundation, Addis Ababa, Ethiopia; LSTM: Liverpool School of Tropical Medicine, UNITED KINGDOM OF GREAT BRITAIN AND NORTHERN IRELAND

## Abstract

**Introduction:**

In Ethiopia, women in remote agrarian and pastoralist regions face major barriers to accessing timely, quality maternal and newborn health (MNH) services, leading to high rates of home births and preventable mortality. This study assessed the implementation of the Village Health Leader (VHL)-led community-based MNH package in vulnerable agrarian and pastoralist communities in Ethiopia.

**Methods:**

Implementation research deploying a mixed-methods process evaluation across seven health centers and 38 vulnerable communities in the Afar, Somali, and Oromia regions was carried out from December 2023 to July 2025. VHLs were trained to deliver community-based MNH interventions that included misoprostol for the prevention of postpartum hemorrhage, chlorhexidine for umbilical cord care, iron-folic acid for the prevention of anemia, and postpartum family planning counseling and services. Quantitative program monitoring data were collected from 3,980 pregnant women and analyzed descriptively. Qualitative data were collected through 90 in-depth interviews and 14 focus group discussions with VHLs, health workers, postpartum women, community members, and supervisors. Qualitative data were coded in ATLAS.ti using both deductive and inductive approaches. Findings from the quantitative and qualitative components were integrated to provide a comprehensive assessment of implementation.

**Results:**

The intervention reached about 4,000 pregnant women, with high rates of uptake for lifesaving MNH services. Among women who gave birth, 35% delivered at home, 92% used misoprostol, and almost all applied chlorhexidine to the umbilical cord stump. Qualitative findings indicated generally positive perceptions of the intervention and highlighted its acceptability and feasibility. VHLs demonstrated strong adherence to protocols and timely referrals to health facilities. Challenges included supply shortages, lack of incentives for VHLs, and weak referral feedback systems. Despite these constraints, the intervention improved access, enhanced equity, and did not disincentivize facility-based deliveries.

**Conclusions:**

Community-based delivery of lifesaving MNH interventions by VHLs is both feasible and acceptable in most underserved communities. The model strengthens the primary health care system by extending its reach, improving the continuum of care, and addressing persistent access barriers. However, sustainability requires reliable supply chains and tailored approaches for pastoralist populations. The findings support the integration of trained community volunteers into national MNH service delivery strategies to reduce preventable maternal and newborn deaths.

## Introduction

Despite substantial investments in maternal and newborn health (MNH), maternal and neonatal mortality remain major public health challenges in Ethiopia [1,2]. The country’s maternal mortality ratio was estimated at 195 deaths per 100,000 live births in 2023, corresponding to approximately 8,000 maternal deaths annually. Hemorrhage remains a major cause of maternal death, accounting for about 56% of 4,530 reviewed maternal deaths [2,3], while postpartum hemorrhage (PPH) contributed to more than a quarter of hospital-based maternal deaths [4]. Neonatal deaths are similarly driven by sepsis (28%), birth asphyxia (27%), and prematurity (18%) [5]. These preventable deaths are particularly concerning in remote agrarian and pastoralist regions, where skilled assistance and timely emergency obstetric and newborn care remain challenging.

Ethiopia’s extensive geographically dispersed agrarian and pastoralist populations, including communities living in mountainous, sparsely populated, and mobile settings where health facilities may be distant and transportation difficult, face additional barriers to facility-based care, including poor transportation, financial hardship, socio-cultural norms, and mistrust of the health system [6,7]. As a result, women and newborns in these settings experience delays in receiving lifesaving interventions, particularly when complications such as PPH, sepsis, hypothermia, and birth asphyxia occur. These challenges underscore the need for community-based strategies that complement facility-based care and expand equitable access to timely, lifesaving MNH interventions [8,9].

Evidence from various low-and middle-income countries (LMICs) demonstrates that community health workers (CHWs) can effectively deliver essential MNH services and commodities when properly trained and supported [10,11]. Community-based interventions such as participatory women’s groups, home visits, and the distribution of targeted MNH supplies have led to improved care practices, increased service uptake, and reduced maternal and neonatal mortality [12,13]. Specifically, evidence from LMICs shows that CHW-led delivery of misoprostol, iron-folic acid (IFA), chlorhexidine, and progestin-only pills (POPs) can improve maternal and newborn health by reducing PPH-related mortality without discouraging facility births, preventing maternal anemia, lowering cord infections and neonatal mortality, and addressing unmet contraceptive needs [14–18]. These interventions therefore represent evidence-based practices with established potential to improve MNH outcomes.

However, the existence of effective interventions does not necessarily ensure their routine use, particularly where access to facility-based care is limited. Despite these successes, scale-up of community-based MNH interventions has been uneven, hindered by policy hesitations, training and supervision gaps, and concerns about misuse or adverse effects [19]. In Ethiopia, while the recent national self-care guideline supports the provision of over-the-counter oral contraceptive pills and the use of misoprostol for abortion care without direct supervision of a healthcare provider, community-based distribution of misoprostol for the prevention of PPH has not yet been formally adopted as national policy. Accordingly, the routine scope of work for community health workers does not include the distribution of misoprostol or POPs. This creates an implementation gap: evidence-based MNH interventions are available, but their delivery through community-based platforms has not been fully integrated into routine services, particularly for women who face barriers to accessing facility-based care.

Addressing this implementation gap requires strategies that can extend evidence-based interventions to households while strengthening linkages with the existing primary health care (PHC) system. To address these gaps, JSI and Amref Health Africa, in collaboration with the Ministry of Health (MOH) and regional health bureaus, conducted implementation research from December 2023 to July 2025 to evaluate a Village Health Leader (VHL)-led strategy for delivering community-based lifesaving MNH interventions. The implementation strategy used VHLs to conduct household visits, identify and follow up pregnant women, provide counselling and referral support, and distribute selected MNH commodities, including IFA, misoprostol, chlorhexidine, and POPs. Thus, VHL-led delivery represented an implementation strategy designed to address barriers to access and support the routine uptake and use of evidence-based MNH interventions at the household and community levels.

The study used a mixed-methods process evaluation to assess how this implementation strategy was delivered within routine PHC settings. Specifically, the evaluation assessed its reach, safety, fidelity, feasibility, acceptability, and integration within the health system. The process evaluation was important for understanding not only whether the interventions reached intended beneficiaries, but also how, under what conditions, and with what implementation challenges the VHL-led model was delivered in vulnerable agrarian and pastoralist communities. The study therefore generated policy-relevant evidence to inform future consideration of community-based distribution of selected lifesaving MNH interventions and potential scale-up and institutionalization of VHL-led community-based MNH service delivery models across Ethiopia and similar contexts.

## Methods

### Context

Ethiopia has made considerable investments in community-based primary health care. In a typical woreda, the PHC system consists of one primary hospital and five Primary Health Care Units (each comprising a health center and about five health posts). Health posts are staffed by two Health Extension Workers (HEWs), linked with health centers, provide essential Health Extension Program (HEP) services—including reproductive, maternal, newborn, and child health (RMNCH) services—at the community level. Community-based volunteers, such as VHLs and other community structures further support health education, identification and follow-up of pregnant women, referral, and continuity of care.

Health centers provide more advanced RMNCH services such as skilled delivery, basic emergency obstetric and newborn care, and management of childhood illnesses. Primary hospitals function as referral hubs, offering comprehensive emergency obstetric and newborn care, inpatient maternal and pediatric care, surgical services, and specialist support. This hierarchical structure creates an important continuum between households, health posts, health centers, and hospitals, within which community-based workers can facilitate early identification, counseling, referral, and follow-up.

VHLs were introduced by the MOH in 2020 as community-selected, gender-mixed volunteers (mostly young women), with at least a grade 6 education who serve as trusted role models. Their introduction builds on Ethiopia’s longer-standing experience with community-based health delivery rather than creating a parallel community health system. They bridge HEWs with households and communities, enhancing service reach and uptake. VHLs support key RMNCH services—antenatal care, postnatal care, family planning, immunization, growth monitoring, and health education—by identifying pregnant women, linking them to care, tracing defaulters, and promoting healthy behaviors (S1 File).

Recruitment is community-based, followed by a 5-day, 24-hour national curriculum training (S2 File) covering maternal and child health, disease prevention, hygiene, communication, community engagement, data management, and field practice. Training is facilitated by health center staff and HEWs to ensure practical competency.

This established community-health infrastructure therefore provided the platform for the present intervention, which sought to extend selected lifesaving MNH interventions to households in vulnerable agrarian and pastoralist communities.

### Intervention description

Since April 2022, the JSI and Amref Health Africa consortium have been implementing the *Improving Primary Health Care Service Delivery* project [20]. As part of this initiative, implementation research developed a VHL-led, community-based lifesaving MNH service delivery model aimed at reaching vulnerable women with limited access to facility-based MNH services in contexts of extreme adversity. Core interventions include: (1) promoting facility births and educating communities on birth preparedness and complication readiness, and (2) facilitating the advance distribution of IFA supplements, misoprostol, chlorhexidine, and progestin-only pills since December 2023.

These interventions are being implemented across seven health centers and 38 vulnerable communities (8 agrarian and 30 pastoral), serving a total population of 210,787. The VHLs conduct home visits to overcome barriers related to geographic distance and limited access to health facilities in Chifra woreda (Afar, pastoral), Degahabur woreda (Somali, pastoral), and Seka Chekorsa woreda (Oromia, agrarian), with JSI leading implementation in agrarian areas and Amref in pastoral settings.

The intervention sites were selected in close collaboration with regional health bureaus and woreda health office experts. Using routine Health Management Information System data on institutional delivery coverage and consultations with woreda health managers, woredas with persistently low coverage were identified for the community-based MNH intervention. For the purposes of this study, “*low facility birth*” was operationally defined as a setting in which fewer than 30% of expected births occurred in a health facility as a pragmatic program-selection criterion. This selection was further supported by a formative household survey, which revealed institutional delivery coverage of 10% in pastoral areas and 17% in the agrarian Seka Chekorsa woreda, while another agrarian woreda with higher facility birth rates (77%) was excluded.

Following a series of consultations with MOH and regional health bureau program managers and co-design processes, stakeholders agreed on a package of lifesaving, community-based MNH interventions to be delivered by VHLs through three scheduled home visits—two antepartum and one postpartum. The VHLs conducted active surveillance to identify pregnant women in their catchment areas and enrolled them during the first home visit. As part of their roles, VHLs not only provided counseling but also distributed selected essential commodities, including misoprostol, chlorohexidine, POPs for postpartum family planning, and IFA supplements. Once enrolled, VHLs monitored the women throughout pregnancy and provided postpartum support, ensuring delivery of essential interventions and guidance on birth preparedness, complication readiness, and other MNH-related services (Fig 1).

**Fig 1:**
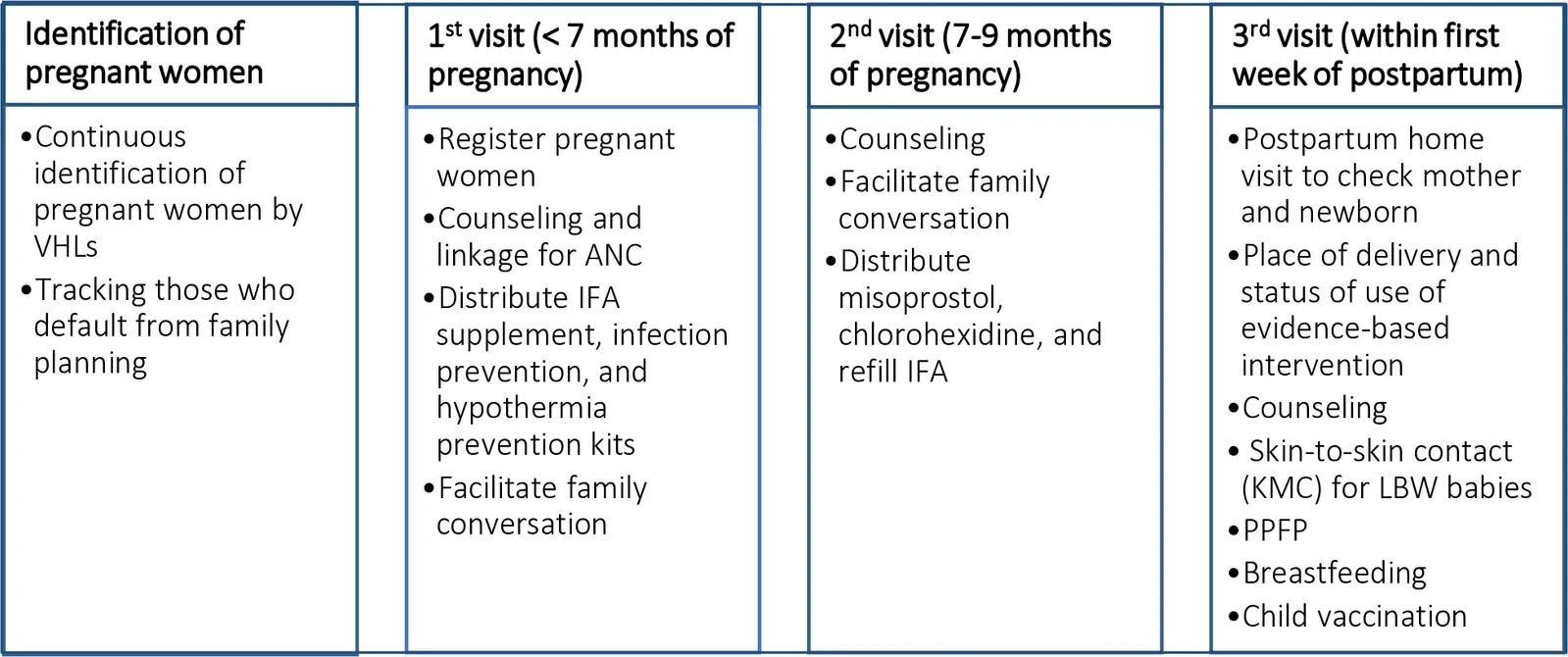
Community-based delivery of MNH services and home visitation schedule for vulnerable communities.

To implement this model, 187 VHLs were recruited and trained—78 in agrarian areas and 109 in pastoralist areas—and provided with start-up kits containing misoprostol, chlorhexidine, POPs, IFA, counseling materials, and data collection tools. In pastoralist areas, kits also included clean and safe birth supplies, such as a towel, cape, soaks, delivery plastic sheet, cord blade, and cord tie, informed by the co-design workshop. The project facilitated VHL recruitment in close collaboration with the woreda health office, health center staff, HEWs, kebele managers, and community representatives. Following recruitment, VHLs received a 5-day basic training, followed by an additional 2-day project-specific training that explicitly covered commodity distribution protocols (including eligibility, dosage, timing, counseling, and referral), as well as smartphone use in agrarian areas (S2 File).

To support these efforts, the project developed several key tools: an implementation guide, an orientation manual for community volunteers, package inserts for mothers, a monitoring tool, and an adverse event reporting tool, all translated into the local language. Additionally, the project secured essential life-saving drugs and supplies from the health system to ensure the availability of evidence-based MNH intervention packages.

As part of strengthening community-based lifesaving MNH interventions, the project has also been strengthening the health system to improve maternity waiting homes and strengthening referral links within the intervention facilities.

### Design and sampling methods

A mixed-method process evaluation was conducted to address the research questions by combining qualitative inquiry with quantitative program monitoring data. The RE-AIM framework (Reach, Effectiveness, Adoption, Implementation, and Maintenance) [21] was used to as the overarching framework to structure the evaluation and to examine the delivery and integration of the intervention within routine PHC settings. Within this overarching framework, Proctor et al.’s implementation outcomes [22] were used to operationalize key aspects of implementation of the strategies (Table 1). For the quantitative component, longitudinal program monitoring data were drawn from a cohort of pregnant women enrolled in the study during the implementation period (n = 3,980) and followed at home by VHLs through three scheduled household contacts: two antenatal contacts during pregnancy and one postpartum contact within the first week after delivery.

**Table 1:** Matrix of study objectives, research questions, and methods.

SN	RE-AIM domain/Implementation outcome	Objective	Measures	Data sources & methods
1	Reach & Effectiveness	Evaluate the reach and safety of community-based delivery mechanisms for lifesaving MNH interventions.	**• Reach:** Home visits by VHLs, distribution and utilization of misoprostol, chlorhexidine, and POPs, measured through mothers’ self-reports to VHLs during home visits and recorded using the District Health Information Software (DHIS2) Tracker system **• Safety:** Unintended consequences (shifts in facility delivery rates, misuse, harm, timing of swallowing misoprostol)	Program monitoring data; IDIs and FGDs
2	Implementation (Fidelity)	Measure the implementation fidelity of community-based delivery of MNH interventions.	**•** Engagement of VHLs **•** Adherence to counseling guidelines and protocols	IDIs and FGDs
3	Implementation (Feasibility & Acceptability)	Explore community perceptions, feasibility, and acceptability of the program.	**•** Perceived relevance and cultural appropriateness of interventions **•** Contextual factors- Feasibility and acceptability of VHL-led service delivery	IDIs and FGDs
4	Implementation & Maintenance (Integration with PHC)	Assess the extent of integration with the PHC system.	**•** VHLs’ interaction with the health system **•** Support systems, coaching, and performance reviews	IDIs and FGDs

The qualitative component adopted a phenomenological study design and employed purposive stratified sampling to explore implementation experiences across diverse perspectives in both agrarian and pastoral settings. Participants included postpartum women (beneficiaries) who had given birth within the previous six months, VHLs (implementers), and program managers such as HEWs, Primary Health Care Unit directors, HEP focal persons, woreda health experts, and project field officers. Theoretical sampling guided participant selection to ensure data richness, with interviews conducted until thematic saturation was reached [18]—typically after a minimum of six in-depth interviews (IDIs) per respondent category [19]. Data collection involved IDIs and focus group discussions (FGDs) using semi-structured guides tailored for each group.

### Data collection

The VHLs collected longitudinal quantitative data from cohort of 3,980 pregnant women, representing all women identified and enrolled by VHLs during the study period between 01/12/2024 to 30/06/2025. Women were followed through home visits, beginning in early pregnancy and continuing through the immediate postpartum period. At each visit, they updated individual records, capturing information on the uptake of lifesaving MNH interventions, medications, and maternal conditions during the intrapartum and postpartum phases. In agrarian areas, VHLs were equipped with smartphones loaded with a customized DHIS2 Tracker tool, enabling direct digital data entry. In contrast, in pastoral areas, VHLs recorded information using paper forms, which were later digitized by HEWs at the health post level into the DHIS2 Tracker system. The indicators captured by VHLs included pregnancy identification and enrolment, completion of scheduled contacts, birth-preparedness and facility-delivery counselling, distribution and reported use of IFA, misoprostol, chlorhexidine, and POPs, place of delivery, referrals, and postpartum follow-up.

Qualitative data were collected through interviews and discussion guides with open-ended questions from informants and group discussants between 03/02/2025 to 21/02/2025. Separate semi-structured guides were used for VHLs, postpartum women, and program managers/health-system actors, with questions tailored to their respective roles and experiences. The guides covered implementation fidelity, reach and accessibility, safety, acceptability, feasibility, supervision, commodity availability, referral, and integration with the primary health care system. VHLs were additionally asked about household visits, identification and enrolment of pregnant women, and adherence to program protocols; postpartum women were asked about their experiences with VHL services, access to and use of MNH interventions, family and community influences, and referral; and program managers were asked about program oversight, VHL–HEW coordination, supervision, resources, and system integration. The guides were informed by the study objectives and evaluation framework, with RE-AIM providing the overarching structure and Proctor et al.’s implementation outcomes informing the key implementation constructs.

Trained qualitative experts conducted interviews and FGDs in natural settings in their preferred languages, Amharic and Afaan Oromo, with conversations audio-recorded using digital recorders after obtaining participants’ consent. Interviews lasted a mean of 25 minutes (range: 15–37 minutes), while FGDs lasted a mean of 86 minutes (range: 60–94 minutes). Audio recordings were transcribed verbatim and translated into English for analysis. Throughout data collection, the IDI and FGD guides (S3 File) were revisited and adjusted as needed to ensure comprehensive information was captured.

### Trustworthiness

To ensure data credibility, open-ended questions were designed to be simple, objective, and non-leading, minimizing bias throughout the research process. Four experienced qualitative research assistants collected the data while maintaining neutrality to avoid influencing participants’ responses. They probed and paraphrased participants to clarify or expand their answers. Detailed documentation—including data collection procedures, field notes, raw data, analysis notes, and interpretation-ensured confirmability. The study employed both IDIs and FGDs on the same phenomena for methodological triangulation. Findings and interpretations were reviewed by the Monitoring, Evaluation, and Research teams of JSI and Amref Health Africa to prevent bias or misinterpretation.

### Data analysis

Quantitative data collected through the DHIS2 Tracker were exported for analysis. Longitudinal records were checked for completeness, duplicate records, inconsistent values, and inconsistencies across repeated contacts. Data cleaning, management, and analysis were conducted using Stata version 15. Individual-level records were organized chronologically according to women’s enrolment dates and subsequent VHL contacts. The longitudinal data were then collapsed by visit frequency and administrative region using Stata’s “*collapse”* command to calculate the frequencies and proportions of the outcome variables.

Qualitative data were analyzed using deductive and inductive thematic analysis. ATLAS.ti was used for data organization, coding, and management. Two Monitoring, Evaluation, and Research team members from JSI and Amref Health Africa jointly reviewed the transcripts and developed a preliminary coding framework based on the study objectives and data collection guides. We discussed emerging concepts and refined the codes as needed. Related codes were then grouped into broader categories and themes through joint review and discussion. Findings were triangulated across participant groups to identify areas of convergence and divergence and enhance the trustworthiness of the analysis. Illustrative quotations were selected to support the identified themes. The qualitative and quantitative methods were integrated during the interpretation phase to provide a comprehensive description of the study findings and produce the final report.

### Ethics

Ethical approval for this study was obtained from the Ethiopian Public Health Association’s Research Ethics Review Committee (Ref. #: EPHA/OG/728/23, July 17, 2023; renewed Ref. #: EPHA/OG/466/24, August 07, 2024). Written permission to conduct the study was also secured from regional health bureaus, zonal health departments, and other relevant institutions.

For the qualitative component, informed written consent was obtained from all participants. They were informed about the purpose of the study, potential benefits and risks, and their right to withdraw or decline to answer any questions at any time. Participation was entirely voluntary, and interviews were conducted only after consent was documented. All data were de-identified before analysis, and strict measures were taken to ensure confidentiality and protect participants’ privacy. The quantitative data analyzed were originally collected for program monitoring through the DHIS2 system, which does not capture personal identifiers. The study was conducted in full compliance with the principles of the Declaration of Helsinki.

## Results

### Qualitative respondent characteristics

The study included a total of 90 individual in-depth interview participants—45 each from agrarian and pastoral communities. These comprised 14 VHLs, 17 HEWs, 31 postpartum mothers (both home and facility deliveries), 10 husbands, 3 mothers-in-law, and 15 supervisors (including project coordinators, HEW supervisors, health center directors, and woreda health office experts). Fourteen FGDs—nine agrarian and five pastoral—with VHLs (4–12 participants each) were also conducted to capture community-level perspectives (Table 2).

**Table 2:** Respondent characteristics.

Respondents	Agrarian	Pastoral	Total
VHLs	7	7	14
HEWs	7	10	17
Postpartum mothers	13	18	31
Supervisors	5	10	15
Husbands/mothers-in-law	13	0	13
Total	45	45	90

### Reach

The program demonstrated strong reach by VHLs, who surmounted physical and infrastructural challenges to identify and engage pregnant women through regular home visits, collaboration with community members, and leveraging local knowledge to ensure no one was missed for timely care and essential medications. This contributed to improved maternal and newborn outcomes and promoted skilled antepartum and intrapartum care.

Study participants generally described VHLs as increasing the reach of MNH services, particularly by identifying pregnant women, conducting household visits, providing essential medicines and counselling, and linking women with health facilities. Some health-center respondents reported that VHLs were reaching most or all mothers in their assigned kebeles.

*After the initiation of the project, many positive changes were observed, including a significant increase in facility-based deliveries, partly due to the strong commitment of the VHLs.* —Supervisor, IDI, Pastoral.

Between December 2023 and June 2025, a cohort of 3,980 pregnant women—2,439 from agrarian communities and 1,541 from pastoral communities—representing 50% of the expected pregnancies (75% in agrarian areas and 33% in pastoral areas), was followed across 38 kebeles. The first antepartum home visit was received by 65% of agrarian and 100% of pastoral women, while coverage for the second antepartum visit dropped to 55% and 63%, respectively. The third postpartum visit reached 45% of agrarian and 54% of pastoral women (Table 3).

**Table 3:** Coverage of antepartum and postpartum home visits among pregnant women enrolled in the study, December 2023–June 2025.

Visit type	Agrarian (n = 2,439)	Pastoral (n = 1,541)	Total (n = 3,980)
First antepartum visit	1,591 (65%)	1,541 (100%)	3,132 (79%)
Second antepartum visit	1,330 (55%)	977 (63%)	2,307 (58%)
Third postpartum visit	1,086 (45%)	827 (54%)	1,913 (48%)

The project’s longitudinal monitoring data, collected through mothers’ self-reports to VHLs during home visits and recorded using the DHIS2 Tracker system, showed that among the 1,867 women who gave birth during the intervention period, 38% in agrarian and 31% in pastoral areas delivered at a health facility. Among those who gave birth at home, 92% used misoprostol—89% in agrarian and 97% in pastoral settings. Similarly, 77% of women in agrarian and 89% in pastoral areas applied chlorhexidine to their newborns’ umbilical cords. However, only one-third (29%) of women received postpartum progesterone-only pills from VHLs—49% in agrarian areas and 16% in pastoral areas (Table 4).

**Table 4:** Uptake of MNH services among pregnant women enrolled in the study, December 2023–June 2025.

Variable	Agrarian, n (%)	Pastoral, n (%)	Total, n (%)
Gave birth	1,040	827	1,867
Home delivery	396 (38.1)	256 (31.0)	652 (34.9)
Used misoprostol (among home births)	352 (88.9)	248 (96.9)	600 (92.0)
Applied chlorohexidine	801 (77.1)	736 (89.0)	1,537 (82.3)
Received progesterone-only pills	510 (49.0)	132 (15.9)	642 (34.4)

However, these positive assessments were not universal. Participants from particularly remote areas described substantial difficulties in reaching women because of long distances, poor roads, dispersed settlements, and limited transportation. In one health-post catchment area, facility staff reported that women predominantly delivered at home because the health center was too far away.

Referral and emergency transportation emerged as a major barrier across participant groups. The VHLs described remaining with women during labor and contacting health facilities for assistance but reported difficulty obtaining ambulances because of poor roads and limited ambulance availability. Health-center staff similarly described delayed ambulance responses as a persistent constraint on timely referral, particularly for obstetric emergencies.

### Safety

According to routine incident and adverse event monitoring by VHLs, HEWs, and project field staff, no adverse outcomes, including maternal or newborn deaths or misuse of misoprostol, have been reported in connection with the distribution of the life-saving package.

Qualitative findings were broadly consistent with these monitoring data. Most participants described the medicines as acceptable and reported no major problems with their use. However, a small number of participants reported side effects from the medications, which did not affect adherence. For example, one husband reported*:* “the medication caused a burning sensation in her abdomen… but I also emphasized the importance of continuing.” (Husband, IDI, Agrarian).

Community members, especially mothers, expressed gratitude for the interventions provided by VHLs, including life-saving medicines. A VHL explained that “mothers are happy with the medications; many now ask for these pills themselves and report that they experienced less bleeding and abdominal cramping (Malla) after taking the misoprostol” (VHL, FGD, Agrarian).

### Implementation fidelity

**Home visits and service delivery:** VHLs demonstrated strong engagement in MNH service delivery through structured and regular home visits. They provided a continuum of care, from antenatal counseling to postnatal follow-ups, consistently reinforcing key health messages around birth preparedness and complication readiness, facility-based delivery, and postpartum care. Their visits also included health education on newborn care, safe delivery practices, and environmental sanitation.

*We conduct first, second, and third rounds of visits for pregnant women… we provide services within seven days of their delivery, document the birth of the baby, and track their health after birth.* – VHL, IDI, Agrarian.

The VHLs provided hands-on practical instructions on medication use (e.g., misoprostol) and promoted hygiene practices in cases of home delivery. They actively worked to dispel harmful traditional practices through consistent counseling.

*I received medications like misoprostol and chlorhexidine and didn’t face any major challenges in accessing them. I also didn’t encounter any significant difficulties when using them. The instructions provided [by VHLs] were clear and easy to follow.* – Mother, IDI, Pastoral.

**Use of job aids:** VHLs largely reported a good understanding and implementation of the guidelines provided for the delivery of community-based lifesaving MNH interventions. They utilized job aids, visual materials (photos and pictures), simple illustrations, and mobile applications to schedule visits and report services, demonstrating a high level of procedural compliance.

*The manual is not difficult. When we read it, we find that it is helpful and easy to follow.* – VHL, IDI, Agrarian.

However, health-center staff reported variation in VHL proficiency with the mobile application and with adherence to guidelines. Experience-sharing sessions were subsequently used to facilitate peer learning and improve consistency in implementation.

**Implementation challenges:** Despite their commitment and efforts to deliver the lifesaving MNH interventions package, both VHLs and HEWs faced key operational challenges that hindered the effective implementation of the MNH interventions. Across participant groups, the main challenges were not a lack of willingness to implement the intervention but constraints within the wider health-system and community context. These included: competing responsibilities with other community services, supply shortages, and limited recognition. Long travel distances, a nomadic population, and dispersed settlements further complicated the service delivery, making it particularly challenging to identify and enroll pregnant women in remote and hard-to-reach areas.

A key constraint was the frequent shortage of essential medicines and supplies, such as misoprostol and chlorhexidine. These stockouts persisted even when community demand was high, limiting the continuity and effectiveness of care.

*The only problem we have now is a shortage of medicine… Now the community knows the medicine, but there is a shortage.* – HEW, IDI, Agrarian.

*We are currently experiencing a shortage of misoprostol… It is a significant concern.* – VHL, IDI, Pastoral.

In addition, VHLs reported limited recognition from the health system, which affected their motivation. Delays in receiving promised materials like uniforms and badges, coupled with a limited and irregular engagement from the Woreda Health Office, contributed to the VHLs’ sense of neglect. A VHL described that “It is deeply disheartening that we have not received any attention from the Woreda Health Office in the past six months.” (VHL, FGD, Agrarian).

Technical and logistical challenges—such as a lack of reliable power source for charging mobile devices—further hampered VHLs’ capacity to use digital tools for reporting and communication. Some VHLs in agrarian areas mentioned occasional difficulties with technology use or inconsistent use of digital tools, indicating variability in the fidelity of guideline implementation through improved over time.

The VHLs in pastoral communities reported significant challenges in reaching remote and hard-to-reach areas due to geographical barriers, limited transportation, and resource constraints. These factors hindered their ability to provide timely and consistent MNH services. As one informant explained “We are facing challenges such as inadequate access to transportation and cultural barriers.” (VHL, IDI, Pastoral).

### Feasibility and acceptability

**Community acceptance and trust:** VHLs are well accepted and trusted by the community, which has also embraced the community-based delivery of MNH services. The culturally sensitive strategy has further strengthened engagement and trust, ensuring effective implementation.

*The services provided by the VHLs were extremely helpful. Their advice to give birth at a health facility was invaluable, contributing to the health of both me and my newborn.* —Mother, IDI, Pastoral

The services delivered by VHLs were widely accepted and appreciated across communities, with many noting this was the first time such care was available at their doorstep. VHLs are seen as accessible, caring, and deeply embedded in community life, which has enhanced service uptake and compliance. A mother from pastoral community described *“*We all knew the benefits of the intervention, and no one had any reason to object.” (Mother, IDI, Pastoral).

Community members, including women and families, hold a positive view of both the interventions and the role of VHLs. Through consistent awareness efforts, VHLs have built strong trust and rapport, strengthening the link between pregnant women and health facilities.

*Family members and the broader community strongly support women in accessing modern healthcare. There is widespread awareness about its benefits, leading to full acceptance and support.* —Mother, IDI, Pastoral.

A VHL from pastoral areas reflected on community trust:

*The community sees that I play a positive role supporting women, and they trust me because I always tell the truth. I explain the services clearly and share hopes for better comprehensive care.* —VHL, IDI, Pastoral.

**Culture influence:** Cultural and religious beliefs continued to shape some attitudes toward MNH interventions, particularly family planning. In some communities, religious doctrines discouraged contraceptive use, while gender dynamics influenced provider preference, with many women favoring female VHLs for comfort and privacy. Many women preferred female VHLs because they felt more comfortable discussing sensitive issues with other women. As one mother explained, “Many women… prefer being served by female VHLs.” (Mother, IDI, Agrarian). Similarly, a husband noted that “Female VHLs are more effective… women are more comfortable confiding in other women.” (Husband, IDI, Agrarian).

A key informant from the community noted some initial opposition from community members, including religious leaders, over family planning kits included in the packages. As one mother-in-law explained, “Some religions… consider family planning forbidden...” (Mother-in-law, IDI, Agrarian).

### Integration with the PHC system

**Referral links:** The qualitative findings indicated that VHLs were increasingly functioning as a link between households and the formal PHC system. VHLs played a vital role in linking pregnant women to health facilities by initiating referrals, facilitating emergency transport services, and following up at home after facility deliveries. These efforts enhanced continuity of care and improved outcomes but were sometimes undermined by systemic issues such as ambulance delays and inadequate feedback mechanisms from higher-level facilities. Interviewees highlighted that community interventions are well aligned with health posts and centers, with VHLs and HEWs collaborating effectively.

*She received a support letter and gave birth at the health center. They even visited after delivery.* —Husband, IDI, Agrarian.

Participants confirmed a strong partnership between VHLs and health center staff and HEWs, which builds community trust and confidence in the health system.

*The relationship between VHLs and HEW is a promising bridge between the health system and community, as they evaluate their work monthly. While VHL reports its progress to HEWs, the latter reports to the health center.* —Supervisor, IDI, Pastoral.

However, referral system challenges persist, including transportation shortages and long distances between some clusters and health facilities. A supervisor highlighted that “Limited resources, such as ambulances, have affected service quality and implementation.” (Supervisor, IDI, Pastoral).

**Support system, coaching, and performance reviews:** VHLs reported feeling supported by the health system through training and guidance from HEWs and supervisors but identified gaps in the regularity of support and access to resources and ongoing training opportunities.

*We sometimes face shortages of essential items like brochures, registration cards, and materials for awareness-raising. Access to updated program information is also limited. Ongoing training is needed.* —VHL, IDI, Pastoral.

## Discussion

This implementation research provides compelling evidence that the community-based delivery of lifesaving MNH interventions is both feasible and acceptable in most underserved agrarian and pastoralist settings. Leveraging trained VHLs to deliver an integrated MNH package, including misoprostol, chlorhexidine, IFA, and postpartum contraceptives, demonstrated high levels of adherence to protocols, reach, and community acceptance. The model successfully addressed persistent barriers to facility-based care, particularly in geographically remote or culturally marginalized communities, and offers a promising approach to advancing health equity, improving outcomes, and strengthening PHC linkages.

The study confirms that VHL-led delivery of MNH services is feasible and can be implemented with high fidelity in settings where professional health workers are scarce and access to health facilities is limited. VHLs consistently adhered to structured protocols, conducted home visits aligned with antenatal and postnatal schedules, and used job aids and digital tools to support care delivery and documentation. These findings align with evidence from other LMICs, where CHWs have successfully implemented similar MNH interventions when provided with adequate training, supervision, and system support [10,13,23–28].

Implementation fidelity varied between agrarian and pastoral settings, reflecting differences in infrastructure, mobility, and resource availability. In agrarian areas, VHLs identified pregnant women through neighbor interactions and benefited from structured supervision and digital tools like DHIS2 Tracker for real-time follow-up. In pastoral areas, identification occurred during the first home visit, but nomadic lifestyles, remote settlements, and reliance on paper-based tools digitized later by HEWs delayed enrollment and reporting. Despite these challenges, VHLs in both contexts showed strong commitment, using culturally appropriate communication to promote safe use of life-saving commodities. However, persistent stockouts, weak supervision, and lack of recognition remain barriers, underscoring the need for context-specific strategies to strengthen community-based MNH service delivery [27,29].

The high level of community trust in VHLs was instrumental in the program’s success. The VHLs were perceived as accessible, trustworthy, and culturally sensitive, which fostered strong community buy-in. The doorstep delivery of MNH services met an expressed need for accessible, respectful care, especially for women in remote and underserved areas. These findings align with global and national evidence that culturally competent CHWs, when integrated into community structures and supported with sustained health education, are more likely to be accepted and effective [9,26–28]. Importantly, initial skepticism—especially around postpartum contraceptive methods—was addressed through tailored community engagement and involvement of male partners and religious leaders. This facilitated behavior change and fostered collective ownership, underscoring the importance of culturally responsive strategies in the uptake of health innovations [30].

This intervention directly addressed long-standing inequities in MNH service access by bringing essential care to women typically excluded from the formal health system. In both agrarian and pastoralist communities, VHLs reached thousands of pregnant women with essential MNH services [14–16,31–33]. Notably, in hard-to-reach areas where health facilities were often inaccessible due to distance, terrain, or lack of transportation, community-based interventions enabled continued care and saved lives. Among women who gave birth at home, over 90% used misoprostol to prevent postpartum hemorrhage, and the majority applied chlorhexidine for umbilical cord care. This level of reach is particularly important given the historically limited coverage of some evidence-based newborn interventions in Ethiopia. For example, a national survey conducted across four regions reported that 46% of newborns received chlorhexidine at any time after birth, while only 34% received it within 24 hours; coverage was substantially lower among births without skilled attendance [34].

Despite targeted efforts by VHLs to promote facility delivery, institutional birth rates remained low in these pastoralist and remote agrarian settings. Although some improvement was observed, facility birth rates remain constrained by persistent structural barriers, such as long distances, difficult terrain, poor road infrastructure, and limited transportation, as well as entrenched sociocultural norms favoring home birth, as documented in this study. These findings suggest that while community-based strategies can improve safe home practices and partially mitigate access barriers, they are insufficient on their own to drive rapid shifts in delivery location without broader health system strengthening and social change interventions.

The intervention package was found to be both effective and safe. Participants reported improved birth preparedness, reduced complications, and safer home deliveries. Routine adverse event monitoring did not identify any significant safety issues, misuse, or unintended consequences, such as facility avoidance for birth. This aligns with previous studies in Ethiopia and elsewhere demonstrating that advanced distribution of misoprostol and chlorhexidine by trained community workers is not only safe but can also improve outcomes without discouraging facility births [14,25,35,36].

While minor side effects were noted, they did not deter use, and community members expressed satisfaction with the outcomes. Spousal and family support further enhanced the effectiveness of the intervention, reinforcing the role of household dynamics in maternal care-seeking behavior [37,38].

A key strength of this intervention was its integration with the existing PHC system. The VHLs complemented HEWs, initiated referrals, and coordinated with health facilities for emergency care and follow-up. This strengthened the continuity of care and reinforced the credibility of the formal health system. However, integration challenges persist. Weak referral feedback mechanisms, lack of transport for emergencies, and limited engagement from Woreda health offices constrained the full realization of a seamless community–facility care continuum. Strengthening these links—through better resource allocation, formal recognition of VHLs, and investment in transport and communication—will be critical to institutionalizing and sustaining this model. The evidence affirms that strong community–health system integration, bolstered by routine coaching, monitoring, and inclusion in health information systems, improves MNH outcomes, program accountability, and system responsiveness [39,40].

Strengths of the study include its mixed-methods design, which triangulated data from routine monitoring, IDIs, and FGDs to offer a comprehensive understanding of the intervention’s fidelity, feasibility, acceptability, reach, and safety. The study was implemented across both agrarian and pastoralist settings, allowing for comparative insights across diverse contexts. In addition, the program was embedded within the existing HEP, which enhanced real-world applicability and scalability of the findings.

However, the study has limitations. First, the use of program monitoring data may have introduced reporting bias or data quality issues, especially in remote areas where digital reporting tools were inconsistently used due to power or connectivity constraints. Second, some indicators relied on participant or VHL reports and may therefore be subject to recall or reporting bias. Third, mothers-in-law were included among qualitative participants, male/partner perspectives were not captured which limits our understanding of gender dynamics, household decision-making, and partner support in these settings. Third, the study did not systematically assess the cost-effectiveness of the intervention, which is critical for informing policy decisions about scale-up. Finally, the evaluation was conducted during the implementation period and therefore provides limited evidence on the long-term sustainability or maintenance of the intervention. Future research should examine the sustainability, cost-effectiveness, and health impact of VHL-led MNH delivery, while examining gender and household decision-making.

## Conclusions

This implementation research demonstrates that delivering essential MNH interventions through trained VHLs is both feasible and acceptable in hard-to-reach agrarian and pastoralist settings. The approach improved access to life-saving care for women giving birth at home, strengthened linkages with the formal health system, and fostered community empowerment through culturally responsive engagement.

With the right systems in place, this model could be scaled and institutionalized nationally to save more lives. Sustaining and expanding the model will require continuous supply availability, effective referral systems, and motivation mechanisms to retain VHLs. Overall, the study provides timely, policy-relevant insights to guide national strategies for reducing maternal and newborn mortality and advancing universal health coverage in marginalized communities.

## Supporting information

S1 File The roles of VHLs in maternal and child health service delivery. This file outlines the roles of VHLs in maternal and child health service delivery, in line with the national scope of work.
(DOCX)

S2 File VHLs’ training curriculum. This file contains the national basic 5-day training curriculum for VHLs, as well as the 2-day project-specific training curriculum.
(DOCX)

S3 File In-depth interview and focus group discussion guides for community-based delivery of MNH interventions. These are the IDI and FGD guides we used to interview the participants in our study.
(DOCX)

S4 File Qualitative transcriptions. This is selected de-identified interview excerpts used for the analysis and report.
(DOCX)

## Abbreviations

CHW: community health worker
DHIS2: district health information software
FGD: focus group discussion
HEP: health extension program
HEW: health extension worker
IDI: in-depth interview
IFA: iron-folic acid
LMICs: low- and middle-income countries
MNH: maternal and newborn health
MOH: Ministry of Health
PHC: primary health care
POPs: progestin-only pills
PPH: postpartum hemorrhage
RMNCH: reproductive, maternal, newborn, and child health
VHL: village health leader
