# Supplementary material for: Community-based delivery of lifesaving maternal and newborn health interventions in vulnerable Ethiopian communities: Lessons from implementation research
Source: PLoS One. 2026 Nov 5;21(11):e0360788. doi: 10.1371/journal.pone.0360788 (PMC13739096; doi:10.1371/journal.pone.0360788)
Supplement: Supplementary file 1 [file pone.0360788.s005.pdf]

Overall, this manuscript addresses a highly relevant topic with clear implications for implementation and policy in low-income and hard-to-reach settings. The intervention focus is timely, and the clarity of the overall idea and written language is commendable. However, the manuscript needs some refinements, particularly in the description of the intervention, methodological transparency, and presentation of quantitative results.

Introduction

The Introduction is generally clear and well-written. No major concerns are noted in this section.

Methods

1) The Methods section would benefit from additional clarification regarding the intervention context and implementation processes. In particular, it would be helpful to explicitly state whether misoprostol and progestin-only pills were approved by the Ministry of Health of Ethiopia for distribution by Village Health Leaders, and, if so, when, with references. This information is important for understanding the policy and regulatory context of the intervention.

2) The description of the intervention would also benefit from greater detail regarding the distribution of medicines. The roles related to distributing misoprostol, progestin-only pills, and IFA supplements are not clearly reflected in Supplementary File S1 (VHL roles) or Supplementary File S2 (training topics), nor are these activities fully described in the intervention description within the Methods section. Including this information would improve transparency and reproducibility, and clarify the scope of VHL's responsibilities and the training they received regarding these interventions.

3) Further methodological clarification is needed regarding how the use of MNH intervention medicine (for example, misoprostol and chlorhexidine) was assessed. The manuscript reports a high proportion of misoprostol use among home deliveries (e.g., 90%) and 100% chlorhexidine use, which appears difficult to reconcile with the lower levels of ANC attendance reported. It would be helpful to clarify whether “receipt” refers to mothers collecting the medication, self-reported use, or any mechanism used to verify actual intake. A brief explanation of this in the Methods section would strengthen methodological transparency.

Results

1) The presentation of quantitative results would benefit from improved clarity and structure. In several instances, percentages are reported without clearly specifying their denominators, which limits interpretability. For example, statements such as “90% of home deliveries received misoprostol,” and similar data presentations, should clearly indicate the underlying population used to calculate the percentage. Ensuring that all percentages are accompanied by clearly defined denominators would improve clarity.

2) In addition, the Results section would be strengthened by adding a more structured table. I suggest Table 2 in the results, stratified by livelihood context (e.g., agrarian and pastoralist communities in columns), with rows including: total number of participants, ANC visits, postpartum visits, place of delivery (institutional vs. home), and receipt of key commodities such as misoprostol, chlorhexidine, progestin-only pills, and IFA supplements. This would allow readers to more easily compare outcomes across settings.

Discussion

The Discussion section could be strengthened by further reflection on the unexpectedly low levels of institutional delivery observed in the study. The reported proportion of facility delivery, for example, 38% in agrarian communities, seems very low despite strong NGO supported implementation. This appears lower than figures commonly reported by the Ministry of Health. A discussion of possible reasons for this would add depth to the interpretation of the findings.

Conclusion

The conclusion appears too long.
