# Supplementary material for: Community-based delivery of lifesaving maternal and newborn health interventions in vulnerable Ethiopian communities: Lessons from implementation research
Source: PLoS One. 2026 Nov 5;21(11):e0360788. doi: 10.1371/journal.pone.0360788 (PMC13739096; doi:10.1371/journal.pone.0360788)
Supplement: Supplementary file 2 [file pone.0360788.s007.pdf]

A point-by-point response to reviewers

PONE-D-25-45507: Community-Based Delivery of Life-saving Maternal and Newborn Health Interventions in Vulnerable Ethiopian Communities: Lessons from Implementation Research

Dear Editor,

We, the authors, would like to thank the reviewer for their valuable comments. Our point-by-point responses to the reviewer and editor are below each comment. We also reviewed to ensure that this manuscript version conforms to the journal style.

Journal Requirements:

When submitting your revision, we need you to address these additional requirements.

1. Please ensure that your manuscript meets PLOS ONE's style requirements, including those for file naming. The PLOS ONE style templates can be found at https://journals.plos.org/plosone/s/file?id=wjVg/PLOSOne_formatting_sample_main_body.pdf and https://journals.plos.org/plosone/s/file?id=ba62/PLOSOne_formatting_sample_title_authors_affiliations.pdf

Response: Thank you for this comment. We have revised the figure and table citations throughout the text and made additional formatting adjustments to ensure that the manuscript conforms to the journal's style guidelines.

2. We note that the grant information you provided in the 'Funding Information' and 'Financial Disclosure' sections do not match.

When you resubmit, please ensure that you provide the correct grant numbers for the awards you received for your study in the 'Funding Information' section.

Response: Thank you for bringing this to our attention. We have revised the Funding Information section to ensure consistency and have provided the correct grant numbers for this project.

3. We note that this data set consists of interview transcripts. Can you please confirm that all participants gave consent for interview transcript to be published?

If they DID provide consent for these transcripts to be published, please also confirm that the transcripts do not contain any potentially identifying information (or let us know if the participants consented to having their personal details published and made publicly available). We consider the following details to be identifying information:

- Names, nicknames, and initials
- Age more specific than round numbers
- GPS coordinates, physical addresses, IP addresses, email addresses
- Information in small sample sizes (e.g. 40 students from X class in X year at X university)
- Specific dates (e.g. visit dates, interview dates)
- ID numbers

Or, if the participants DID NOT provide consent for these transcripts to be published:
- Provide a de-identified version of the data or excerpts of interview responses
- Provide information regarding how these transcripts can be accessed by researchers who meet the criteria for access to confidential data, including:

a) the grounds for restriction
b) the name of the ethics committee, Institutional Review Board, or third-party organization that is imposing sharing restrictions on the data
c) a non-author, institutional point of contact that is able to field data access queries, in the interest of maintaining long-term data accessibility.
d) Any relevant data set names, URLs, DOIs, etc. that an independent researcher would need in order to request your minimal data set.

For further information on sharing data that contains sensitive participant information, please see: https://journals.plos.org/plosone/s/data-availability#loc-human-research-participant-data-and-other-sensitive-data

If there are ethical, legal, or third-party restrictions upon your dataset, you must provide all of the following details (https://journals.plos.org/plosone/s/data-availability#loc-acceptable-data-access-restrictions):
1. A complete description of the dataset
2. The nature of the restrictions upon the data (ethical, legal, or owned by a third party) and the reasoning behind them
3. The full name of the body imposing the restrictions upon your dataset (ethics committee, institution, data access committee, etc)
4. If the data are owned by a third party, confirmation of whether the authors received any special privileges in accessing the data that other researchers would not have
5. Direct, non-author contact information (preferably email) for the body imposing the restrictions upon the data, to which data access requests can be sent

Response: Thank you for your comment. We confirm that all participants provided informed consent for their interview data to be used for research purposes. To ensure confidentiality and protect participant privacy, the data we are submitting have been fully de-identified. All potentially identifying information—including names, nicknames, initials, specific ages, GPS coordinates, addresses, dates, and other contextual

identifiers—has been removed. Excerpts included in the manuscript or supplementary materials are carefully anonymized to prevent identification of individual participants.

4. Please include captions for your Supporting Information files at the end of your manuscript, and update any in-text citations to match accordingly. Please see our Supporting Information guidelines for more information: http://journals.plos.org/plosone/s/supporting-information.

Response: Thank you for your comment. We have added captions for all Supporting Information files at the end of the manuscript and updated the corresponding in-text citations to ensure consistency. The revisions now fully align with the journal's Supporting Information guidelines.

5. If the reviewer comments include a recommendation to cite specific previously published works, please review and evaluate these publications to determine whether they are relevant and should be cited. There is no requirement to cite these works unless the editor has indicated otherwise.

Response: Thank you for this guidance. We confirm that no reviewer comments included recommendations to cite specific previously published works. Therefore, no additional citations were required or added in response to this point.

Reviewers' comments:

Reviewer's Responses to Questions

Reviewer #1: A PDF file with review feedback is attached. I would like to provide the feedback in a separate file which is attached. Overall, this is a manuscripted with relevant ideas and presented in intelligible fashion. Requires a few methodological and quantitative data presentation improvents.

Overall, this manuscript addresses a highly relevant topic with clear implications for implementation and policy in low-income and hard-to-reach settings. The intervention focus is timely, and the clarity of the overall idea and written language is commendable. However, the manuscript needs some refinements, particularly in the description of the intervention, methodological transparency, and presentation of quantitative results.

Introduction

The Introduction is generally clear and well-written. No major concerns are noted in this section.

Response: Thank you for this positive feedback.

Methods

1) The Methods section would benefit from additional clarification regarding the intervention context and implementation processes. In particular, it would be helpful to explicitly state whether misoprostol and progestin-only pills were approved by the Ministry of Health of Ethiopia for distribution by Village Health Leaders, and, if so, when, with references. This information is important for understanding the policy and regulatory context of the intervention.

Response: Thank you for these valuable comments. We have revised the Methods section to provide additional clarification on the policy and regulatory context of the intervention. Specifically, we now explicitly state that, while the recent national self-care guideline supports the provision of over-the-counter oral contraceptive pills and the use of misoprostol for abortion care without direct supervision of a health-care provider, community-based distribution of misoprostol for the prevention of PPH has not yet been formally adopted as national policy. Accordingly, the routine scope of work for Village Health Leaders does not include the distribution of misoprostol or progestin-only pills.

We further clarify that, although community-based distribution of misoprostol has previously been implemented in Ethiopia, it was discontinued due to safety concerns raised by policymakers and program managers. This implementation study was therefore conducted, in collaboration with the Ministry of Health and the National Research Advisory Council, to generate policy-relevant evidence in selected hard-to-reach agrarian and pastoral settings to inform future policy consideration and potential scale-up. ***See Page 4.***

2) The description of the intervention would also benefit from greater detail regarding the distribution of medicines. The roles related to distributing misoprostol, progestin-only pills, and IFA supplements are not clearly reflected in Supplementary File S1 (VHL roles) or Supplementary File S2 (training topics), nor are these activities fully described in the intervention description within the Methods section. Including this information would improve transparency and reproducibility, and clarify the scope of VHL's responsibilities and the training they received regarding these interventions.

Response: Thank you for this important comment. We have revised the manuscript to more clearly describe the roles of VHLs in the distribution of key commodities, including misoprostol, POP, chlorhexidine, and IFA supplements. Specifically, we have updated the Methods section to explicitly outline VHL responsibilities related to eligibility screening, counseling, and distribution of these interventions during home visits. **Pages 7 and 8.**

In addition, the supplementary materials have been revised to improve alignment with the intervention description. The additional two-day project-specific training provided

to VHLs, covering commodity distribution protocols, counseling, and related competencies, has now been incorporated into S2 File.

3) Further methodological clarification is needed regarding how the use of MNH intervention medicine (for example, misoprostol and chlorhexidine) was assessed. The manuscript reports a high proportion of misoprostol use among home deliveries (e.g., 90%) and 100% chlorhexidine use, which appears difficult to reconcile with the lower levels of ANC attendance reported. It would be helpful to clarify whether "receipt" refers to mothers collecting the medication, self-reported use, or any mechanism used to verify actual intake. A brief explanation of this in the Methods section would strengthen methodological transparency.

Response: Thank again you for this insightful comment. We have strengthened the methodological description to improve transparency regarding how the use of MNH intervention commodities was assessed. Specifically, we clarified that these data are derived from the project's longitudinal monitoring system, based on mothers' self-reports to VHLs during routine home visits, and recorded in the DHIS2 Tracker system. Accordingly, "receipt" and "use" refer to self-reported information provided by mothers rather than independently verified consumption.

This clarification has been incorporated in both the Methods and Results sections. **Please see Table 1 on page 9** (Methods) "…. distribution and utilization of misoprostol, chlorhexidine, and POPs, measured through mothers' self-reports to VHLs during home visits and recorded using the DHIS2 Tracker system" and the relevant text on **page 16** (Results), where the following statement has been added: "The project's longitudinal monitoring data, collected through mothers' self-reports to VHLs during home visits and recorded using the DHIS2 Tracker system, showed that …".

Results

1) The presentation of quantitative results would benefit from improved clarity and structure. In several instances, percentages are reported without clearly specifying their denominators, which limits interpretability. For example, statements such as "90% of home deliveries received misoprostol," and similar data presentations, should clearly indicate the underlying population used to calculate the percentage. Ensuring that all percentages are accompanied by clearly defined denominators would improve clarity.

2) In addition, the Results section would be strengthened by adding a more structured table. I suggest Table 2 in the results, stratified by livelihood context (e.g., agrarian and pastoralist communities in columns), with rows including: total number of participants, ANC visits, postpartum visits, place of delivery (institutional vs. home), and receipt of key commodities such as misoprostol, chlorhexidine, progestin-only pills, and IFA supplements. This would allow readers to more easily compare outcomes across settings.

Response: Thank you for these helpful and constructive comments. In response, we have revised the Results section to improve clarity and structure. Specifically, we ensured that all reported percentages are accompanied by clearly defined denominators to enhance interpretability. As such, we have incorporated a new, structured table in the Results section (Table 4) as you suggested, stratified by livelihood context (agrarian and pastoralist communities). The table presents key indicators, including total number of delivered women and home deliveries, and receipt of essential commodities such as misoprostol, chlorhexidine, and progestin-only pills. **Please see Table 4 on page 17.**

Discussion

The Discussion section could be strengthened by further reflection on the unexpectedly low levels of institutional delivery observed in the study. The reported proportion of facility delivery, for example, 38% in agrarian communities, seems very low despite strong NGO supported implementation. This appears lower than figures commonly reported by the Ministry of Health. A discussion of possible reasons for this would add depth to the interpretation of the findings.

Response: Thank you for this insightful comment. We have strengthened the Discussion section by explicitly addressing the relatively low levels of institutional delivery in the study settings. In the revised text, we highlight that despite targeted promotion by VHLs and some improvement, facility birth rates remain constrained by persistent structural barriers as well as entrenched sociocultural norms favoring home birth. As such, the following paragraph has been added in the revsed manuscript, Page 24.

> *"Despite targeted efforts by VHLs to promote facility delivery, institutional birth rates remained low in these pastoralist and remote agrarian settings. Although some improvement was observed, facility birth rates remain constrained by persistent structural barriers, such as long distances, difficult terrain, poor road infrastructure, and limited transportation, as well as entrenched sociocultural norms favoring home birth, as documented in this study. These findings suggest that while community-based strategies can improve safe home practices and partially mitigate access barriers, they are insufficient on their own to drive rapid shifts in delivery location without broader health system strengthening and social change interventions."*

Conclusion

The conclusion appears too long.

Response: Thank you for this helpful comment. We have revised the conclusion to make it more concise and focused, reducing repetition while retaining the key findings

and policy implications.
