# Supplementary material for: Community-based delivery of lifesaving maternal and newborn health interventions in vulnerable Ethiopian communities: Lessons from implementation research
Source: PLoS One. 2026 Nov 5;21(11):e0360788. doi: 10.1371/journal.pone.0360788 (PMC13739096; doi:10.1371/journal.pone.0360788)
Supplement: Supplementary file 3 [file pone.0360788.s001.docx]

S1 File: The roles of VHLs in maternal and child health service delivery

| **Domains** | **Roles in RMNCH** |
| --- | --- |
| ANC, delivery, and PNC | - Identification of pregnant mothers - Health education on danger signs during pregnancy - Birth preparedness and complication readiness - Promote skilled delivery - Nutrition and other care during pregnancy - Family conversation - Promoting postnatal care - Health education on danger signs in newborns |
| Child health | - Awareness creation on common childhood illnesses and management (pneumonia, diarrhea, malaria. - Referral |
| FP | - Promoting family planning - Awareness of the available family planning methods - Referral |
| Immunization | - Identification of zero-dose and under-immunized children - Participate in campaigns - Facilitate outreach and community mobilization |
| Nutrition | - Promoting breastfeeding and complementary feeding - Promote growth monitoring |
