# Supplementary material for: Community-based delivery of lifesaving maternal and newborn health interventions in vulnerable Ethiopian communities: Lessons from implementation research
Source: PLoS One. 2026 Nov 5;21(11):e0360788. doi: 10.1371/journal.pone.0360788 (PMC13739096; doi:10.1371/journal.pone.0360788)
Supplement: Supplementary file 4 [file pone.0360788.s002.docx]

*S2 File. VHLs’ training curriculum*

*Table 1: VHLs basic training topics and national curriculum at recruitment*

| **SN** | **Training topics** | **Time in hrs.** | **Percent** |
| --- | --- | --- | --- |
| 1 | Maternal and child health (family planning, ANC, facility birth, PNC, newborn, child health, immunization, nutrition) | 9 | 26 |
| 2 | Communicable and non-communicable disease prevention and control | 5 | 15 |
| 3 | Hygiene and Sanitation | 5 | 15 |
| 4 | Communication, concept and the roles of the VHLs | 4 | 12 |
| 5 | Working with other community structures- Male, Women Development Groups, youths | 3 | 9 |
| 6 | Data management | 4 | 12 |
| 7 | Field practices | 4 | 12 |
|  | **Total time in hours** | **24** | **100** |

*Table 2: VHLs community-based delivery of MNH interventions training topics*

| **SN** | **Training topics** | **Time** |
| --- | --- | --- |
| 1 | Life-saving health package for mothers and newborns | 30 minutes |
| 2 | Identification of pregnant women | 45 minutes |
| 3 | Key messages for the first round of house-to-house visits and medications provided to mothers | 2 hours |
| 4 | Key messages for the second round of house-to-house visits and medications and ointments provided to mothers | 3 hours 30 minutes |
| 5 | Key messages for the third round of house-to-house visits and supplements provided to mothers | 2 hours 45 minutes |
| 6 | Data collection and management | 1 hour 30 minutes |
|  | **Total time in hours** | **11 hours** |
