# Supplementary material for: Community-based delivery of lifesaving maternal and newborn health interventions in vulnerable Ethiopian communities: Lessons from implementation research
Source: PLoS One. 2026 Nov 5;21(11):e0360788. doi: 10.1371/journal.pone.0360788 (PMC13739096; doi:10.1371/journal.pone.0360788)
Supplement: Supplementary file 5 [file pone.0360788.s003.docx]

# S2 File: IDI and FGD guides for community-based delivery of MNH interventions

|  | **Identification** | |
| --- | --- | --- |
| 1 | Questionnaire ID | ____________________________ |
| 2 | Area Identification | ____________________________ |
| 3 | Name of Woreda | ____________________________ |
| 4 | Name of facility | ____________________________________ |
| 5 | Name of Kebele |  |
| 6 | Name of moderator | _______________________________ |
| 7 | Name of note-taker | _________________________________ |
| 8 | Date of interview/discussion | _______________________________ |
| 9 | Start time: | ______:________ |
| 10 | End time: | ____:______ |

| **Demographic characteristics of informants** |  |
| --- | --- |
| Age |  |
| Sex |  |
| Informant/discussant’s role | - Beneficiaries (mother receiving the interventions - Implementers (VHLs) - Program managers (woreda experts, PHCU directors, HEP focal person, project field officers, HEWs) |

## **IDI and FGD guides for VHLs**

| **SN** | **Main questions** | **Follow-up/probing questions** |
| --- | --- | --- |
| **I** | **Implementation fidelity** |  |
| 1 | Can you describe your typical day or activities as a VHL? | What specific tasks do you prioritize during home visits? |
| 2 | How do you do to ensure all pregnant women are visited as scheduled? |  |
| 3 | How do you ensure adherence to counseling guidelines and protocols? | Have there been instances where you found the guidelines unclear or challenging to follow? |
| 4 | What challenges have you encountered in following the planned activities? And how have you overcome these challenges? | Probe for time, resources, or community resistance |
| **II** | **Reach, accessibility, and safety** |  |
| 5 | What strategies have you used to identify and enroll pregnant women in your catchment? | How do you gain the trust of pregnant women and their families? |
| 6 | Are there groups of women who are harder to reach? If so, why? | - Are there geographical, financial, or cultural barriers? - What solutions have you implemented to address these barriers? |
| 7 | How accessible do you think the life-saving MNH interventions are to women? |  |
| 8 | Have you encountered any safety concerns or unintended consequences? | Can you describe specific incidents (e.g., misuse of medications, resistance to facility delivery)? |
| **III** | **Community perceptions and acceptability** |  |
| 9 | How do women and families perceive these interventions and your role as a VHL? |  |
| 10 | Are there any common misconceptions or concerns raised by the community? How do you address these concerns? |  |
| **IV** | **Integration with the PHC system** |  |
| 11 | How well are you supported by the health system? | What type of feedback or support do you receive from HEWs or supervisors? |
| 12 | Are there gaps in training, resources, or communication? |  |
| 13 | Are there any gaps in the supply chain that affect your ability to deliver care? | Which supplies are often unavailable or delayed? |
| 14 | How does this affect your work or the community’s trust in the program? |  |

## **IDI guides for postpartum mothers**

|  | **Main questions** | **Follow-up/probing questions** |
| --- | --- | --- |
| **I** | **Implementation fidelity** |  |
| 1 | Can you describe your interactions with the VHLs? | How often do they visit you? |
| 2 | What kind of information or support do they provide during home visits? |  |
| 3 | What kinds of support, advice, or care did they provide during your pregnancy? | Did they discuss birth preparedness, complication readiness, or newborn care? |
| 4 | Did you find the information easy to understand and follow? |  |
| **II** | **Reach, accessibility, and safety** |  |
| 5 | Were you able to receive and use the medications provided? | Were there any challenges in accessing or using misoprostol, chlorhexidine, or other supplies? |
| 6 | How did you feel about using these medications? |  |
| 7 | Did you experience any challenges while using the medications or interventions? | Were there any side effects or concerns? |
| **III** | **Community perceptions and acceptability** |  |
| 8 | What do you think about the health services provided by the VHLs? | Do you think their visits were helpful? Why or why not? |
| 9 | Are there areas where you feel their support could be improved? |  |
| 10 | Are there any practices or beliefs in your community that affect the use of these services? | How do cultural or religious beliefs influence decisions about pregnancy and childbirth? |
| 11 | How do family members or community leaders influence these decisions? |  |
| **IV** | **Integration with the PHC system** |  |
| 12 | Did you receive support from health facilities during your pregnancy or delivery? | Were you encouraged or referred to give birth at a facility from VHLs? |
| 13 | What were the main reasons for choosing your delivery location? |  |

## **IDI guides for program managers**

| **SN** | **Main questions** | **Follow-up/probing questions** |
| --- | --- | --- |
| **I** | **Implementation fidelity** |  |
| 1 | Can you describe your role in overseeing the implementation of MNH interventions? | What are your key responsibilities? |
| 2 | How do you coordinate with VHLs and HEWs to ensure smooth implementation? |  |
| 3 | How do you ensure that VHLs and HEWs adhere to project guidelines? | Are there regular performance reviews or coaching sessions? |
| 4 | What challenges have you observed in maintaining adherence to protocols? |  |
| **II** | **Reach, accessibility, and safety** |  |
| 5 | How effective has the program been in reaching the target population? What strategies have been used to address these gaps? | Are there any groups or areas that are harder to reach? |
| 6 | Have there been any reported safety concerns or unintended consequences? How were these issues addressed? | Can you provide examples of specific incidents? |
| **III** | **Community perceptions and acceptability** |  |
| 7 | How has the community responded to the interventions? | Are there differences in acceptance among various groups |
| 8 | What strategies have been used to improve community acceptability? |  |
| **IV** | **Integration with the PHC system** |  |
| 9 | How well are the community-based interventions aligned with the broader health system? | How do VHLs and HEWs interact with health facilities? |
| 10 | What gaps or challenges exist in the referral and reporting systems? |  |
| 11 | Are there gaps in resource availability that affect implementation? | Which resources are most critical but often unavailable? |
| 12 | How do these gaps impact the program's outcomes? |  |
