# Supplementary material for: Community-based delivery of lifesaving maternal and newborn health interventions in vulnerable Ethiopian communities: Lessons from implementation research
Source: PLoS One. 2026 Nov 5;21(11):e0360788. doi: 10.1371/journal.pone.0360788 (PMC13739096; doi:10.1371/journal.pone.0360788)
Supplement: Supplementary file 6 [file pone.0360788.s004.docx]

# S4 File: Selected de-identified interview excerpts

## Theme 1: Reach

Participant: Village Health Leader (VHL)

*“After going from home to home, we identify the pregnant mothers, we then provide them iron tablet during the first phase. We advise them to have follow up from health extension workers. […] We will set up a schedule to reach all the mothers […] However, it is sometimes difficult to reach all the mothers and provide the necessary follow up during the rain as the roads are not suitable.”*

“By making visits, we go from home to home one by one and identify pregnant women and register them and then ask a woman how old she is, how many months pregnant she is. Then tell her the precautions to take from the time she becomes pregnant.”

*“We do visits to pregnant women based on a scheduled basis which is in turns. During the visits we will give all the advice a woman needs.”*

Participant: Expert/program manager

*“It was unthinkable to deliver iron in a number of hard-to-reach areas until we established this VHLs system. Our coverage for iron uptake has increased tremendously […] If there were no VHLs in our hard to reach areas, it would be very difficult to render the service.”*

Participant: Expert/program manager

*“The VHLs have things they have to deliver to a mother. […] They provide the Iron tablet, check the package whether the mother used it or not. They tell her about the importance of the Misoprostol she is supposed to swallow after delivery. […] They also check the health status of the baby.”*

Participant: Expert/program manager

*“When they identify pregnant women, they send that mother to a health center. […] These VHLs have brought many changes not only on maternal and newborn health but also on other health issues. They are sending mothers for family planning, child immunization, antenatal care and postnatal care. In a critical case when it is a must to deliver at home, they provide the medicine.”*

## Theme 2: Safety

Participant: HEW

**“***There are some mothers who say it has caused burning. I have told them as it is very important both for her and the baby.”*

Participant: Expert/program manager

*“We have received no reports of incidents related to side effects from those areas so far. We have been using our own formats for such reports, as there are different side effects for different medications.”*

Participant: HEW

*“Yes. There are some mothers who say it has caused burning. I have told them as it is very important both for her and the baby.”*

Participant: VHL

*“Yes. For example, if we have medicine shortage, we inform the health extension worker. We will also tell the program coordinator. They bring and deliver it to us when we tell them we have shortage of medicine. They are also helping us when we have any challenges. They are coming from the health center and supervising us.”*

*“There is no big challenge but we have a problem related to medicine. We are facing problems with misoprostol.”*

Participant: Expert/program manager

*“Initially we were trying to check its impact in terms of its side effect. But we weren’t able to monitor it and find it. We informed the supervisors and directors to follow anything that can happen including the side effects and inappropriate use. […] I haven’t received any complaints so far.”*

## Theme 3: Implementation fidelity and operational challenges

Participant: VHL

*“The activities listed in the guidelines/manuals developed for maternal and child health is being implemented very well. Moreover, once a week we have been having necessary discussions with the health extension workers on maternal and child health.”*

Participant: HEW

*“I am the only one, but the population is more than [10,000], it is difficult for one health extension worker to reach all of them. There is a place which takes you [a long time] to reach. […] However, I am making the VHLs reach out and bring the report.”*

Participant: Expert/program manager

*“The first problem is related to the ambulance service. When a pregnant woman is sick and about to give birth, she often finds it very difficult to reach the health center immediately due to lack of ambulances.”*

Participant: VHL

*“Since there is no electricity in our area and there is a big problem with charging our phones. This has caused us a lot of problems, including when we have to call each other for discussions.”*

Participant: HEW

*“Yes, it is not available at the health center. The program manager had been officially writing a letter and asking for it. Previously, we were receiving all of the medicine together. But we are not getting it now.”*

Participant: HEW

*“The first problem is related to an ambulance. Once there was a mother in labour. I tried their phone multiple times but it was not working. […] There is a big problem with the ambulance service.”*

Participant: Expert/program manager

*“The main problem is transportation. If transportation is available, we can do more. Additionally, the frequency of provision of training is taking longer. As long as they are not getting training, the service they provide can be decreased. This thing should also be solved.”*

Participant: Expert/program manager

*“During our visit we observe as they are implementing the activity based on the guidelines. They are conducting first, second and third round visits. We also have review meeting at Health center. […] They also have a meeting at health post level to evaluate performance of each other.”*

## Theme 4: Feasibility and acceptability

Participant: VHL

*“The community is happy with it and they are asking for continuation of these activities. They were saying previously our mothers were not willing to go health center for services, now they are getting the services at home.”*

*“They are very happy. Pregnant mothers are especially happy. They are happy to get the misoprostol and chlorhexidine that is applied on the baby’s umbilicus. Previously they had been receiving iron tablets from health facilities after travelling long distances. Now they are getting it at home. They call and ask for it when they finish the one they have collected. They are very happy. Now they are sad due to the shortage of medicine. They are asking why the provision of the drug was discontinued.”*

Participant: Mother

*“I feel more comfortable sharing my problems openly with female village health leaders (VHLs). I find it difficult to discuss personal or sensitive matters with male VHLs. Women tend to connect more easily with other women, making it easier to discuss private issues.”*

*“I feel more comfortable sharing my problems openly with female village health leaders. I find it difficult to discuss personal or sensitive matters with male village health leaders. Women tend to connect more easily with other women, making it easier to discuss private issues.”*

Participant: Mother

*“The services provided by the village health leaders (VHLs) were extremely helpful. Their advice to give birth at a health facility was invaluable, contributing to the health of both myself and my newborn.”*

## Theme 5: Integration with the primary health care system

Participant: Expert/program manager

*“They will call me and inform me. I then called for the ambulance.”*

Participant: Expert/program manager

*“It is aligned. Maternal and newborn health activities are the services that are provided during and after pregnancy. They are helping the mother to deliver at a health facility. They are engaged in carrying and transporting pregnant mothers who are in need of support.”*

*“Regarding the referral, I always get feedback from the health center. I am not getting any feedback from the hospital.”*

*“The problem is related to an ambulance. Once there was a mother in labor. I tried their phone multiple times, but it was not working. I then called the office, but they were not sending an ambulance. I then called the program manager and the ambulance came fast.”*
