# Supplementary material for: Community-based delivery of lifesaving maternal and newborn health interventions in vulnerable Ethiopian communities: Lessons from implementation research
Source: PLoS One. 2026 Nov 5;21(11):e0360788. doi: 10.1371/journal.pone.0360788 (PMC13739096; doi:10.1371/journal.pone.0360788)
Supplement: Supplementary file 7 [file pone.0360788.s008.docx]

**A point-by-point response to reviewers**

PONE-D-25-45507R1: Community-Based Delivery of Life-saving Maternal and Newborn Health Interventions in Vulnerable Ethiopian Communities: Lessons from Implementation Research

Dear Editor,

We, the authors, would like to thank yourself and the reviewers for their valuable comments. Our point-by-point responses to the reviewer and editor are below each comment. We also reviewed to ensure that this manuscript version conforms to the journal style.

Many thanks to the authors for this interesting and well-written manuscript. I have a number of relatively minor revisions suggested below, the most pressing of which are methodological, specifically around providing detail about the analytical processes undertaken. After these are addressed I am happy to see this accepted.

**Response:** Thank you for the positive and encouraging comments. We have addressed all comments, with particular attention to providing greater detail and clarity on the analytical processes. These revisions have strengthened the methodological transparency and overall quality of the manuscript.

**Abstract**

-It is more appropriate to simply call the study design  a “implementation research, deploying a mixed methods process evaluation” as an “implementation research process evaluation” is not really a study design.

Response: Thank you for this clarification. We have revised the sentence to describe the study as implementation research deploying a mixed-methods process evaluation.

-In your methods description, please add a sentence about your analysis beyond just triangulation.

Response: Revised as suggested to provide additional detail on the analytical approach beyond triangulation.

-Please list the domains in the same order in which they are described in the methods in the main text for better flow.

Response: Revised as suggested to present the domains in the same order as in the Methods section.

**Introduction**

-Please quantify the burden of PPH deaths and some of the other conditions you noted where possible to highlight the extent of the problem.

**Response**: Thank you for the valuable feedback. We have quantified the burden of MNH mortality and PPH deaths in the revised manuscript.

-Please provide a bit more detail on the unique features of the Ethiopian geography/health system that make CHWs there so important.

**Response:** Thank you for these important comments. We have expanded the Introduction section to describe Ethiopia’s geographically dispersed agrarian and pastoralist populations, barriers related to distance, mobility, and access to health facilities, and the structure of the primary healthcare system.

-Please provide a bit more background around the role of CHWs in Ethiopia, as they are considerably more entrenched than in other healthcare systems, and that is an important contextual factor in interpreting your results.

Response: We provided additional background on the HEP/ HEWs and community-based volunteers in linking households with the formal health system. Finally, we clarify that the VHLs build on this existing community health platform rather than representing a stand-alone community health worker program in the **Context** section

-To really couch this as implementation research, it would be important to note that there are clearly evidence-based practice (e.g. misoprostol for PPH prevention), but there are gaps in their implementation due to the absence of uptake of facility-based care. Hence there is a need to test implementation strategies to support the routine use of misoprostol as needed, and VHL distribution of misoprostol at the household level was one such strategy that was evaluated. Basically, please highlight the evidence-based practices explicitly, the implementation strategy (i.e. VHL-led MNH care including home visits and distribution of misoprostol, IFA, chlorhexidine, and POP), and the importance of their evaluation, specifically via process evaluation.

**Response:** Thank you for this important comment. We have revised the **Introduction** to clarify that the intervention package and delivery strategies.

**Methods**

-Please define the cutoff to be determined as “low” for facility births

**Response**: We have revised the Methods section to define low facility-delivery coverage as fewer than 30% of expected births occurring in a health facility as a pragmatic program-selection criterion used to identify communities with particularly low uptake of facility-based childbirth and a substantial need for community-based MNH interventions.

-Please explain why RE-AIM was used/how it mapped to the process evaluation domains, which go beyond those in RE-AIM.

**Response**: We have clarified that RE-AIM served as the overarching framework for structuring the evaluation, while Proctor et al.’s implementation outcomes were used to operationalize the key implementation constructs.

-Program monitoring data is part of the process evaluation. Please in Table 1 remove where you have “process evaluation” and the brackets and just rite “IDIs, FGDs”.

Response: Revised as suggested. Table 1 now lists IDIs and FGDs without the additional “process evaluation” designation.

-The “quantitative component” is not clear. Please provide more detail on exactly what this is (presumably what’s in tables 3 and 4? What is “antepartum”? Why not “antenatal” as is more commonly uses? It’s also not clear why only the first two ANC visits were measured and the first PNC visit? It would be important to see justification for the specific focus on these indicators). Please indicate a sample size (3980?) calculation for this component. Please provide more detail about what specifically what indicators were captured by VHLs.

**Response**: Thank you for this detailed comment. We have expanded the Methods section to describe the quantitative component as a longitudinal program monitoring cohort of 3,980 eligible pregnant women enrolled during the implementation period and followed by VHLs through three scheduled household contacts: two antenatal contacts during pregnancy and one postpartum contact within the first week after delivery.

We have also detailed the indicators captured by VHLs, including pregnancy identification and enrolment, completion of scheduled contacts, birth-preparedness and facility-delivery counselling, distribution and reported use of IFA, misoprostol, chlorhexidine and POP, place of delivery, referrals, and postpartum follow-up.

-What did the interview and FGD guides include? Please describe these according to each participant type, as presumably they were asked different things. Was any literature used to inform them?

**Response**: We have expanded the Methods section to describe the qualitative data collection tools and their development. Separate semi-structured guides were used for VHLs, postpartum women, and programme managers/health-system actors, with questions tailored to their respective roles and covering implementation fidelity, reach, safety, acceptability, feasibility, and integration within the primary healthcare system. The guides were informed by the study objectives and evaluation framework, with RE-AIM providing the overarching structure and Proctor’s implementation outcomes informing the key implementation constructs.

-What language were interviews and FGDs carried out in? Was translation necessary (please describe the process of translation if so)?

**Response**: Interviews and FGDs were conducted in participants’ preferred languages, Amharic and Afaan Oromo, by trained interviewers. Audio recordings were transcribed verbatim and translated into English for analysis.

-How long did interviews and FGDs last?

**Response**: Interviews lasted a mean of 25 minutes (range: 15–37 minutes), while FGDs lasted a mean of 86 minutes (range: 60–94 minutes).

-There is no detail on analysis provided. Please explain in more detail how qualitative analysis was undertaken, and what quantitative analysis was carried out.

**Response**: We have revised the Methods section to provide greater detail on both qualitative and quantitative analyses, including the analytical processes used beyond triangulation.

**Results**

-Please ensure that results are presented in the same order in which the domains appear in Table 1 for better flow.

**Response**: Revised. The Implementation Fidelity section has been moved to follow Safety, consistent with the order of domains in Table 1.

-Just about the section on “safety”, please provide a representative quotation in support of the paragraph about qualitative findings.

**Response:** Thank you for this suggestion. We have added a representative quotation to the Safety subsection illustrating participants’ reports of medication-related side effects while indicating that these did not result in treatment discontinuation.

-It’s not quite clear if there were any findings that were unique to a specific participant group, or if there were any divergent views worth flagging? For 90 interviews/FGDs, it’s quite a small amount of qualitative data presented. Are findings as comprehensive as they could be?

**Response**: We also revisited the qualitative dataset to make participant-group-specific findings and divergent views more explicit. The revised Results distinguish findings reported by VHLs, HEWs, health-centre staff, mothers, and other community/family participants and, where relevant, differences between agrarian and pastoral settings.

-It is unclear what results (if any?) stem from “program monitoring”. Please remove mention of this in the methods and discussion if it was not presented here. If it was, please make this more clear. If program monitoring was the quantitative survey, that needs to be made much more clear.

**Response**: We have clarified that the programme monitoring data constituted the quantitative longitudinal cohort data collected as part of routine programme implementation. The Methods, Results, and Discussion have been revised accordingly to make this explicit.

**Discussion**

-It would be important to understand the reach of the evidence-based practices in the absence of VHL delivery. For instance, roughly how widespread is the use of chlorhexidine?

**Response**: Thank you for this suggestion. We have revised the Discussion to provide greater clarity on the reach and use of the evidence-based practices in the absence of VHL delivery, including chlorhexidine.

-The discussion describes “weak supervision”, but this is not a finding that is emphasised in the results.

**Response**: We have revised the Discussion to ensure that the interpretation of supervision is consistent with the findings presented in the Results.

-There are more limitations around the approach taken—including qualitative data collection—that can be addressed. For instance, no males/partner perspectives in the pastoralist communities.

-It would also be useful to highlight more where your study team feels future research needs to be directed.

**Response**: Thank you for these important comments. We have expanded the Limitations section to acknowledge that, although husbands/partners and mothers-in-law were included among qualitative participants, male/partner perspectives were not captured in the pastoralist communities. And further research is also needed to determine whether improved implementation reach translates into sustained use of evidence-based MNH interventions and improved maternal and newborn outcomes.

**Typographically**

-Throughout, please use “healthcare” instead of “health-care”

-You don’t need to introduce IFA as an acronym in the abstract as it’s not repeated in the abstract.

-Please don’t introduce an acronym unless it is repeated multiple times—otherwise please just write the name in-full (e.g. IPHCSD, HMIS, PHC)

-If you do use an acronym, please use it consistently (e.g. VHL, PPH, and FGD are introduced, but in the results and discussion are sometimes written in-full).

-Please remove all quotation marks from the quotations in the results section that are two sentences or longer. Quotations should only be indented and italicised when they are two sentences or longer. If they are shorter than two sentences, they are not italicised, and should be integrated into the paragraph (not indented) and DO then have quotation marks.

Response: Thank you for identifying these editorial and typographical issues. We have reviewed the manuscript and addressed the comments throughout. Specifically, we have standardized “healthcare”, removed unnecessary acronyms, ensured that retained acronyms are used consistently, and revised the formatting and punctuation of quotations according to the journal’s guidelines.

**Review Comments to the Author**
I noticed a minor error, possibly a typographical one, in Table 4 on page 17. Specifically, the percentages in the “Total” column for the rows “Home delivery” and “Used misoprostol” (69.0% and 92.0%, respectively) appear to be incorrect and should be revised.

**Response:** Thank you for identifying this error. The percentages were typographical errors and have been corrected in the revised Table 4.
