# Supplementary material for: Community-based delivery of lifesaving maternal and newborn health interventions in vulnerable Ethiopian communities: Lessons from implementation research
Source: PLoS One. 2026 Nov 5;21(11):e0360788. doi: 10.1371/journal.pone.0360788 (PMC13739096; doi:10.1371/journal.pone.0360788)
Supplement: Supplementary file 8 [file pone.0360788.s009.docx]

**A point-by-point response to reviewers**

PONE-D-25-45507R2: Community-Based Delivery of Life-saving Maternal and Newborn Health Interventions in Vulnerable Ethiopian Communities: Lessons from Implementation Research

Additional Editor Comments:

Many thanks to the authors for this timely revision. Apologies for the back-and-forth, but I am still not quite satisfied with how analysis is described. There are also a few typographical edits outstanding.

**Response:** Thank you for your continued guidance and constructive feedback. We have carefully addressed the remaining comments and made the necessary revisions to improve the data analysis and correct the outstanding typographical issues.

-Abstract: In the introduction, it should be Village Health Leader (VHL)-led (not leaders). You write “village health leaders” in full in the methods—please use VHL. Also in the methods, please change the text of the first sentence to “Implementation research deploying a mixed-methods process evaluation across seven health centers and 30 vulnerable communities in the Afar, Somali, and Oromia regions was carried out from December 2023 to July 2025.”

**Response**: Thank you for this helpful clarification. We have made the requested revisions in the Abstract and Methods.

-Please double-check all acronyms throughout for consistency (VHL is introduced, then “village health leader” is still used sometimes (e.g. top of page 8 and throughout)), including removing acronyms that are not repeated.

**Response**: Thank you for highlighting this. We have reviewed the manuscript for consistency in the use of acronyms and terminology. We now use VHL consistently after its first definition and have removed acronyms that were used only once or were not necessary.

-Analysis is still inadequately described.

- Please have two separate sections: one for data collection and one for data analysis.

**Response**: Thank you for this comment. We have separated the Data Collection and Data Analysis sections. The revised Data Analysis section now provides a detailed description of the analytical approaches used for both the quantitative and qualitative data, see below.

- Coding using ATLAS.ti is not the analytical approach—ATLAS.ti is merely software. Was this a framework analysis? How many people worked on the qualitative analysis? Was any kind of coding framework established, and how? What were the actual steps taken, moving from having a transcript in-hand to moving to the actual sections of the results and the representative quotations. How did this happen? This needs to be very clear. You might consider looking at the COREQ guidelines here that outline some key considerations when reporting on qualitative analysis (as well as reporting data collection, etc.): [https://onlinelibrary.wiley.com/pb-assets/assets/17416612/COREQ_Checklist-1556513515737.pdf](https://track.editorialmanager.com/CL0/https:%2F%2Fonlinelibrary.wiley.com%2Fpb-assets%2Fassets%2F17416612%2FCOREQ_Checklist-1556513515737.pdf/1/010f01a0d78a9213-17c3dd4e-be28-4f7a-a0ab-20eb44e856a5-000000/Ks1Wt01b-TjGwP4giJwheltr1qkZ3NaNHYwP2_OawK8=258)

**Response**: Thank you for this important clarification. We have revised the manuscript to clearly distinguish the analytical approach from the software used. The revised manuscript presented as follows,

*“Qualitative data were analyzed using deductive and inductive thematic analysis. ATLAS.ti was used for data organization, coding, and management. Two Monitoring, Evaluation, and Research team members from JSI and Amref Health Africa jointly reviewed the transcripts and developed a preliminary coding framework based on the study objectives and data collection guides. We discussed emerging concepts and refined the codes as needed. Related codes were then grouped into broader categories and themes through joint review and discussion. Findings were triangulated across participant groups to identify areas of convergence and divergence and enhance the trustworthiness of the analysis. Illustrative quotations were selected to support the identified themes. The qualitative and quantitative methods were integrated during the interpretation phase to provide a comprehensive description of the study findings and produce the final report.”*

- It’s also not clear to the reader how you move from longitudinal data to the results. Did you need to clean the data in any way? How were these analysed? Using what software? What sort of values did you create and how?

**Response**: Thank you for this comment. We have expanded the description of quantitative data management and analysis. The revised manuscript presented as follows,

*“Quantitative data collected through the DHIS2 Tracker were exported for analysis. Longitudinal records were checked for completeness, duplicate records, inconsistent values, and inconsistencies across repeated contacts. Data cleaning, management, and analysis were conducted using Stata version 15. Individual-level records were organized chronologically according to women’s enrolment dates and subsequent VHL contacts. The longitudinal data were then collapsed by visit frequency and administrative region using Stata’s “collapse” command to calculate the frequencies and proportions of the outcome variables.”*

- It’s also not quite clear why it is 3980 women. I presume that this simply represents *all* of the women that VHLs engaged with during the study period? Please make this more explicit in the section on data collection.

**Response**: Thank you for highlighting this. We have clarified in the Data Collection section that the 3,980 women represent the full number of pregnant women identified and enrolled by VHLs during the study period.
