# Supplementary material for: Community-based delivery of lifesaving maternal and newborn health interventions in vulnerable Ethiopian communities: Lessons from implementation research
Source: PLoS One. 2026 Nov 5;21(11):e0360788. doi: 10.1371/journal.pone.0360788 (PMC13739096; doi:10.1371/journal.pone.0360788)
Supplement: Peer review history

# Peer review: Decision Letter 0

**DOI:** 10.1371/journal.pone.0360788.r001

**Authors:** Emma Campbell

19 Mar 2026

Dear Dr. Tiruneh,

Thank you for submitting your manuscript to PLOS ONE. After careful consideration, we feel that it has merit but does not fully meet PLOS ONE’s publication criteria as it currently stands. Therefore, we invite you to submit a revised version of the manuscript that addresses the points raised during the review process.

Please submit your revised manuscript by May 03 2026 11:59PM. If you will need more time than this to complete your revisions, please reply to this message or contact the journal office at plosone@plos.org. When you're ready to submit your revision, log on to https://www.editorialmanager.com/pone/ and select the 'Submissions Needing Revision' folder to locate your manuscript file.

- A letter that responds to each point raised by the academic editor and reviewer(s). You should upload this letter as a separate file labeled 'Response to Reviewers'.
- A marked-up copy of your manuscript that highlights changes made to the original version. You should upload this as a separate file labeled 'Revised Manuscript with Track Changes'.
- An unmarked version of your revised paper without tracked changes. You should upload this as a separate file labeled 'Manuscript'.

If you would like to make changes to your financial disclosure, please include your updated statement in your cover letter. Guidelines for resubmitting your figure files are available below the reviewer comments at the end of this letter.

If applicable, we recommend that you deposit your laboratory protocols in protocols.io to enhance the reproducibility of your results. Protocols.io assigns your protocol its own identifier (DOI) so that it can be cited independently in the future. For instructions see: https://journals.plos.org/plosone/s/submission-guidelines#loc-laboratory-protocols. Additionally, PLOS ONE offers an option for publishing peer-reviewed Lab Protocol articles, which describe protocols hosted on protocols.io. Read more information on sharing protocols at https://plos.org/protocols?utm_medium=editorial-email&utm_source=authorletters&utm_campaign=protocols.

We look forward to receiving your revised manuscript.

Kind regards,

Emma Campbell, Ph.D

Staff Editor

PLOS One

Journal Requirements:

When submitting your revision, we need you to address these additional requirements.

1. Please ensure that your manuscript meets PLOS ONE's style requirements, including those for file naming. The PLOS ONE style templates can be found at

https://journals.plos.org/plosone/s/file?id=wjVg/PLOSOne_formatting_sample_main_body.pdf and

https://journals.plos.org/plosone/s/file?id=ba62/PLOSOne_formatting_sample_title_authors_affiliations.pdf

2. We note that the grant information you provided in the ‘Funding Information’ and ‘Financial Disclosure’ sections do not match.

When you resubmit, please ensure that you provide the correct grant numbers for the awards you received for your study in the ‘Funding Information’ section.

3. We note that this data set consists of interview transcripts. Can you please confirm that all participants gave consent for interview transcript to be published?

If they DID provide consent for these transcripts to be published, please also confirm that the transcripts do not contain any potentially identifying information (or let us know if the participants consented to having their personal details published and made publicly available). We consider the following details to be identifying information:

- Names, nicknames, and initials

- Age more specific than round numbers

- GPS coordinates, physical addresses, IP addresses, email addresses

- Information in small sample sizes (e.g. 40 students from X class in X year at X university)

- Specific dates (e.g. visit dates, interview dates)

- ID numbers

Or, if the participants DID NOT provide consent for these transcripts to be published:

- Provide a de-identified version of the data or excerpts of interview responses

- Provide information regarding how these transcripts can be accessed by researchers who meet the criteria for access to confidential data, including:

a) the grounds for restriction

b) the name of the ethics committee, Institutional Review Board, or third-party organization that is imposing sharing restrictions on the data

c) a non-author, institutional point of contact that is able to field data access queries, in the interest of maintaining long-term data accessibility.

d) Any relevant data set names, URLs, DOIs, etc. that an independent researcher would need in order to request your minimal data set.

For further information on sharing data that contains sensitive participant information, please see: https://journals.plos.org/plosone/s/data-availability#loc-human-research-participant-data-and-other-sensitive-data

If there are ethical, legal, or third-party restrictions upon your dataset, you must provide all of the following details (https://journals.plos.org/plosone/s/data-availability#loc-acceptable-data-access-restrictions):

1. A complete description of the dataset

2. The nature of the restrictions upon the data (ethical, legal, or owned by a third party) and the reasoning behind them

3. The full name of the body imposing the restrictions upon your dataset (ethics committee, institution, data access committee, etc)

4. If the data are owned by a third party, confirmation of whether the authors received any special privileges in accessing the data that other researchers would not have

5. Direct, non-author contact information (preferably email) for the body imposing the restrictions upon the data, to which data access requests can be sent

4. Please include captions for your Supporting Information files at the end of your manuscript, and update any in-text citations to match accordingly. Please see our Supporting Information guidelines for more information: http://journals.plos.org/plosone/s/supporting-information.

5. If the reviewer comments include a recommendation to cite specific previously published works, please review and evaluate these publications to determine whether they are relevant and should be cited. There is no requirement to cite these works unless the editor has indicated otherwise.

[Note: HTML markup is below. Please do not edit.]

Reviewers' comments:

Reviewer's Responses to Questions

**Comments to the Author**

1. Is the manuscript technically sound, and do the data support the conclusions?

Reviewer #1: Yes

**********

2. Has the statistical analysis been performed appropriately and rigorously?

Reviewer #1: No

**********

3. Have the authors made all data underlying the findings in their manuscript fully available?

The PLOS Data policy

Reviewer #1: Yes

**********

4. Is the manuscript presented in an intelligible fashion and written in standard English?

Reviewer #1: Yes

**********

Reviewer #1: A PDF file with review feedback is attached. I would like to provide the feedback in a separate file which is attached. Overall, this is a manuscripted with relevant ideas and presented in intelligible fashion. Requires a few methodological and quantitative data presentation improvents.

**********

what does this mean?). If published, this will include your full peer review and any attached files.

If you choose “no”, your identity will remain anonymous but your review may still be made public.

**Do you want your identity to be public for this peer review?**  For information about this choice, including consent withdrawal, please see our Privacy Policy

Reviewer #1: **Yes:**  Yaliso Yaya

**********

[NOTE: If reviewer comments were submitted as an attachment file, they will be attached to this email and accessible via the submission site. Please log into your account, locate the manuscript record, and check for the action link "View Attachments". If this link does not appear, there are no attachment files.]

To ensure your figures meet our technical requirements, please review our figure guidelines: https://journals.plos.org/plosone/s/figures

You may also use PLOS’s free figure tool, NAAS, to help you prepare publication quality figures: https://journals.plos.org/plosone/s/figures#loc-tools-for-figure-preparation.

NAAS will assess whether your figures meet our technical requirements by comparing each figure against our figure specifications.

Attachment Submitted filename: Review feedback PONE-D-25-45507.pdf

# Author comment: Author response to Decision Letter 1

**DOI:** 10.1371/journal.pone.0360788.r002

27 Mar 2026

A point-by-point response to reviewers

PONE-D-25-45507: Community-Based Delivery of Life-saving Maternal and Newborn Health Interventions in Vulnerable Ethiopian Communities: Lessons from Implementation Research

Dear Editor,

We, the authors, would like to thank the reviewer for their valuable comments. Our point-by-point responses to the reviewer and editor are below each comment. We also reviewed to ensure that this manuscript version conforms to the journal style.

Journal Requirements:

When submitting your revision, we need you to address these additional requirements.

1. Please ensure that your manuscript meets PLOS ONE's style requirements, including those for file naming. The PLOS ONE style templates can be found at

https://journals.plos.org/plosone/s/file?id=wjVg/PLOSOne_formatting_sample_main_body.pdf and

https://journals.plos.org/plosone/s/file?id=ba62/PLOSOne_formatting_sample_title_authors_affiliations.pdf

Response: Thank you for this comment. We have revised the figure and table citations throughout the text and made additional formatting adjustments to ensure that the manuscript conforms to the journal’s style guidelines.

2. We note that the grant information you provided in the ‘Funding Information’ and ‘Financial Disclosure’ sections do not match.

When you resubmit, please ensure that you provide the correct grant numbers for the awards you received for your study in the ‘Funding Information’ section.

Response: Thank you for bringing this to our attention. We have revised the Funding Information section to ensure consistency and have provided the correct grant numbers for this project.

3. We note that this data set consists of interview transcripts. Can you please confirm that all participants gave consent for interview transcript to be published?

If they DID provide consent for these transcripts to be published, please also confirm that the transcripts do not contain any potentially identifying information (or let us know if the participants consented to having their personal details published and made publicly available). We consider the following details to be identifying information:

- Names, nicknames, and initials

- Age more specific than round numbers

- GPS coordinates, physical addresses, IP addresses, email addresses

- Information in small sample sizes (e.g. 40 students from X class in X year at X university)

- Specific dates (e.g. visit dates, interview dates)

- ID numbers

Or, if the participants DID NOT provide consent for these transcripts to be published:

- Provide a de-identified version of the data or excerpts of interview responses

- Provide information regarding how these transcripts can be accessed by researchers who meet the criteria for access to confidential data, including:

a) the grounds for restriction

b) the name of the ethics committee, Institutional Review Board, or third-party organization that is imposing sharing restrictions on the data

c) a non-author, institutional point of contact that is able to field data access queries, in the interest of maintaining long-term data accessibility.

d) Any relevant data set names, URLs, DOIs, etc. that an independent researcher would need in order to request your minimal data set.

For further information on sharing data that contains sensitive participant information, please see: https://journals.plos.org/plosone/s/data-availability#loc-human-research-participant-data-and-other-sensitive-data

If there are ethical, legal, or third-party restrictions upon your dataset, you must provide all of the following details (https://journals.plos.org/plosone/s/data-availability#loc-acceptable-data-access-restrictions):

1. A complete description of the dataset

2. The nature of the restrictions upon the data (ethical, legal, or owned by a third party) and the reasoning behind them

3. The full name of the body imposing the restrictions upon your dataset (ethics committee, institution, data access committee, etc)

4. If the data are owned by a third party, confirmation of whether the authors received any special privileges in accessing the data that other researchers would not have

5. Direct, non-author contact information (preferably email) for the body imposing the restrictions upon the data, to which data access requests can be sent

Response: Thank you for your comment. We confirm that all participants provided informed consent for their interview data to be used for research purposes. To ensure confidentiality and protect participant privacy, the data we are submitting have been fully de-identified. All potentially identifying information—including names, nicknames, initials, specific ages, GPS coordinates, addresses, dates, and other contextual identifiers—has been removed. Excerpts included in the manuscript or supplementary materials are carefully anonymized to prevent identification of individual participants.

4. Please include captions for your Supporting Information files at the end of your manuscript, and update any in-text citations to match accordingly. Please see our Supporting Information guidelines for more information: http://journals.plos.org/plosone/s/supporting-information.

Response: Thank you for your comment. We have added captions for all Supporting Information files at the end of the manuscript and updated the corresponding in-text citations to ensure consistency. The revisions now fully align with the journal’s Supporting Information guidelines.

5. If the reviewer comments include a recommendation to cite specific previously published works, please review and evaluate these publications to determine whether they are relevant and should be cited. There is no requirement to cite these works unless the editor has indicated otherwise.

Response: Thank you for this guidance. We confirm that no reviewer comments included recommendations to cite specific previously published works. Therefore, no additional citations were required or added in response to this point.

Reviewers' comments:

Reviewer's Responses to Questions

Reviewer #1: A PDF file with review feedback is attached. I would like to provide the feedback in a separate file which is attached. Overall, this is a manuscripted with relevant ideas and presented in intelligible fashion. Requires a few methodological and quantitative data presentation improvents.

Overall, this manuscript addresses a highly relevant topic with clear implications for implementation and policy in low-income and hard-to-reach settings. The intervention focus is timely, and the clarity of the overall idea and written language is commendable. However, the manuscript needs some refinements, particularly in the description of the intervention, methodological transparency, and presentation of quantitative results.

Introduction

The Introduction is generally clear and well-written. No major concerns are noted in this section.

Response: Thank you for this positive feedback.

Methods

1) The Methods section would benefit from additional clarification regarding the intervention context and implementation processes. In particular, it would be helpful to explicitly state whether misoprostol and progestin-only pills were approved by the Ministry of Health of Ethiopia for distribution by Village Health Leaders, and, if so, when, with references. This information is important for understanding the policy and regulatory context of the intervention.

Response: Thank you for these valuable comments. We have revised the Methods section to provide additional clarification on the policy and regulatory context of the intervention. Specifically, we now explicitly state that, while the recent national self-care guideline supports the provision of over-the-counter oral contraceptive pills and the use of misoprostol for abortion care without direct supervision of a health-care provider, community-based distribution of misoprostol for the prevention of PPH has not yet been formally adopted as national policy. Accordingly, the routine scope of work for Village Health Leaders does not include the distribution of misoprostol or progestin-only pills.

We further clarify that, although community-based distribution of misoprostol has previously been implemented in Ethiopia, it was discontinued due to safety concerns raised by policymakers and program managers. This implementation study was therefore conducted, in collaboration with the Ministry of Health and the National Research Advisory Council, to generate policy-relevant evidence in selected hard-to-reach agrarian and pastoral settings to inform future policy consideration and potential scale-up. See Page 4.

2) The description of the intervention would also benefit from greater detail regarding the distribution of medicines. The roles related to distributing misoprostol, progestin-only pills, and IFA supplements are not clearly reflected in Supplementary File S1 (VHL roles) or Supplementary File S2 (training topics), nor are these activities fully described in the intervention description within the Methods section. Including this information would improve transparency and reproducibility, and clarify the scope of VHL's responsibilities and the training they received regarding these interventions.

Response: Thank you for this important comment. We have revised the manuscript to more clearly describe the roles of VHLs in the distribution of key commodities, including misoprostol, POP, chlorhexidine, and IFA supplements. Specifically, we have updated the Methods section to explicitly outline VHL responsibilities related to eligibility screening, counseling, and distribution of these interventions during home visits. Pages 7 and 8.

In addition, the supplementary materials have been revised to improve alignment with the intervention description. The additional two-day project-specific training provided to VHLs, covering commodity distribution protocols, counseling, and related competencies, has now been incorporated into S2 File.

3) Further methodological clarification is needed regarding how the use of MNH intervention medicine (for example, misoprostol and chlorhexidine) was assessed. The manuscript reports a high proportion of misoprostol use among home deliveries (e.g., 90%) and 100% chlorhexidine use, which appears difficult to reconcile with the lower levels of ANC attendance reported. It would be helpful to clarify whether “receipt” refers to mothers collecting the medication, self-reported use, or any mechanism used to verify actual intake. A brief explanation of this in the Methods section would strengthen methodological transparency.

Response: Thank again you for this insightful comment. We have strengthened the methodological description to improve transparency regarding how the use of MNH intervention commodities was assessed. Specifically, we clarified that these data are derived from the project’s longitudinal monitoring system, based on mothers’ self-reports to VHLs during routine home visits, and recorded in the DHIS2 Tracker system. Accordingly, “receipt” and “use” refer to self-reported information provided by mothers rather than independently verified consumption.

This clarification has been incorporated in both the Methods and Results sections. Please see Table 1 on page 9 (Methods) “.... distribution and utilization of misoprostol, chlorhexidine, and POPs, measured through mothers’ self-reports to VHLs during home visits and recorded using the DHIS2 Tracker system” and the relevant text on page 16 (Results), where the following statement has been added: “The project’s longitudinal monitoring data, collected through mothers’ self-reports to VHLs during home visits and recorded using the DHIS2 Tracker system, showed that …”.

Results

1) The presentation of quantitative results would benefit from improved clarity and structure. In several instances, percentages are reported without clearly specifying their denominators, which limits interpretability. For example, statements such as “90% of home deliveries received misoprostol,” and similar data presentations, should clearly indicate the underlying population used to calculate the percentage. Ensuring that all percentages are accompanied by clearly defined denominators would improve clarity.

2) In addition, the Results section would be strengthened by adding a more structured table. I suggest Table 2 in the results, stratified by livelihood context (e.g., agrarian and pastoralist communities in columns), with rows including: total number of participants, ANC visits, postpartum visits, place of delivery (institutional vs. home), and receipt of key commodities such as misoprostol, chlorhexidine, progestin-only pills, and IFA supplements. This would allow readers to more easily compare outcomes across settings.

Response: Thank you for these helpful and constructive comments. In response, we have revised the Results section to improve clarity and structure. Specifically, we ensured that all reported percentages are accompanied by clearly defined denominators to enhance interpretability. As such, we have incorporated a new, structured table in the Results section (Table 4) as you suggested, stratified by livelihood context (agrarian and pastoralist communities). The table presents key indicators, including total number of delivered women and home deliveries, and receipt of essential commodities such as misoprostol, chlorhexidine, and progestin-only pills. Please see Table 4 on page 17.

Discussion

The Discussion section could be strengthened by further reflection on the unexpectedly low levels of institutional delivery observed in the study. The reported proportion of facility delivery, for example, 38% in agrarian communities, seems very low despite strong NGO supported implementation. This appears lower than figures commonly reported by the Ministry of Health. A discussion of possible reasons for this would add depth to the interpretation of the findings.

Response: Thank you for this insightful comment. We have strengthened the Discussion section by explicitly addressing the relatively low levels of institutional delivery in the study settings. In the revised text, we highlight that despite targeted promotion by VHLs and some improvement, facility birth rates remain constrained by persistent structural barriers as well as entrenched sociocultural norms favoring home birth. As such, the following paragraph has been added in the revsed manuscript, Page 24.

“Despite targeted efforts by VHLs to promote facility delivery, institutional birth rates remained low in these pastoralist and remote agrarian settings. Although some improvement was observed, facility birth rates remain constrained by persistent structural barriers, such as long distances, difficult terrain, poor road infrastructure, and limited transportation, as well as entrenched sociocultural norms favoring home birth, as documented in this study. These findings suggest that while community-based strategies can improve safe home practices and partially mitigate access barriers, they are insufficient on their own to drive rapid shifts in delivery location without broader health system strengthening and social change interventions.”

Conclusion

The conclusion appears too long.

Response: Thank you for this helpful comment. We have revised the conclusion to make it more concise and focused, reducing repetition while retaining the key findings and policy implications.

Attachment Submitted filename: Point-by-point response reviewers and editor.pdf

# Peer review: Decision Letter 1

**DOI:** 10.1371/journal.pone.0360788.r003

**Authors:** Tara Tancred

13 Sep 2026

PLOS One

Dear Dr. Tiruneh,

Thank you for submitting your manuscript to PLOS One. After careful consideration, we feel that it has merit but does not fully meet PLOS One’s publication criteria as it currently stands. Therefore, we invite you to submit a revised version of the manuscript that addresses the points raised during the review process.

==============================

Many thanks to the authors for this interesting and well-written manuscript. I have a number of relatively minor revisions suggested below, the most pressing of which are methodological, specifically around providing detail about the analytical processes undertaken. After these are addressed I am happy to see this accepted.

Abstract

-It is more appropriate to simply call the study design  a “implementation research, deploying a mixed methods process evaluation” as an “implementation research process evaluation” is not really a study design.

-In your methods description, please add a sentence about your analysis beyond just triangulation.

-Please list the domains in the same order in which they are described in the methods in the main text for better flow.

Introduction

-Please quantify the burden of PPH deaths and some of the other conditions you noted where possible to highlight the extent of the problem.

-Please provide a bit more detail on the unique features of the Ethiopian geography/health system that make CHWs there so important.

-Please provide a bit more background around the role of CHWs in Ethiopia, as they are considerably more entrenched than in other healthcare systems, and that is an important contextual factor in interpreting your results.

-To really couch this as implementation research, it would be important to note that there are clearly evidence-based practice (e.g. misoprostol for PPH prevention), but there are gaps in their implementation due to the absence of uptake of facility-based care. Hence there is a need to test implementation strategies to support the routine use of misoprostol as needed, and VHL distribution of misoprostol at the household level was one such strategy that was evaluated. Basically, please highlight the evidence-based practices explicitly, the implementation strategy (i.e. VHL-led MNH care including home visits and distribution of misoprostol, IFA, chlorhexidine, and POP), and the importance of their evaluation, specifically via process evaluation.

Methods

-Please define the cutoff to be determined as “low” for facility births

-Please explain why RE-AIM was used/how it mapped to the process evaluation domains, which go beyond those in RE-AIM.

-Program monitoring data is part of the process evaluation. Please in Table 1 remove where you have “process evaluation” and the brackets and just rite “IDIs, FGDs”.

-The “quantitative component” is not clear. Please provide more detail on exactly what this is (presumably what’s in tables 3 and 4? What is “antepartum”? Why not “antenatal” as is more commonly uses? It’s also not clear why only the first two ANC visits were measured and the first PNC visit? It would be important to see justification for the specific focus on these indicators). Please indicate a sample size (3980?) calculation for this component. Please provide more detail about what specifically what indicators were captured by VHLs.

-What did the interview and FGD guides include? Please describe these according to each participant type, as presumably they were asked different things. Was any literature used to inform them?

-What language were interviews and FGDs carried out in? Was translation necessary (please describe the process of translation if so)?

-How long did interviews and FGDs last?

-There is no detail on analysis provided. Please explain in more detail how qualitative analysis was undertaken, and what quantitative analysis was carried out.

Results

-Please ensure that results are presented in the same order in which the domains appear in Table 1 for better flow.

-Just about the section on “safety”, please provide a representative quotation in support of the paragraph about qualitative findings.

-It’s not quite clear if there were any findings that were unique to a specific participant group, or if there were any divergent views worth flagging? For 90 interviews/FGDs, it’s quite a small amount of qualitative data presented. Are findings as comprehensive as they could be?

-It is unclear what results (if any?) stem from “program monitoring”. Please remove mention of this in the methods and discussion if it was not presented here. If it was, please make this more clear. If program monitoring was the quantitative survey, that needs to be made much more clear.

Discussion

-It would be important to understand the reach of the evidence-based practices in the absence of VHL delivery. For instance, roughly how widespread is the use of chlorhexidine?

-The discussion describes “weak supervision”, but this is not a finding that is emphasised in the results.

-There are more limitations around the approach taken—including qualitative data collection—that can be addressed. For instance, no males/partner perspectives in the pastoralist communities.

-It would also be useful to highlight more where your study team feels future research needs to be directed.

Typographically

-Throughout, please use “healthcare” instead of “health-care”

-You don’t need to introduce IFA as an acronym in the abstract as it’s not repeated in the abstract.

-Please don’t introduce an acronym unless it is repeated multiple times—otherwise please just write the name in-full (e.g. IPHCSD, HMIS, PHC)

-If you do use an acronym, please use it consistently (e.g. VHL, PPH, and FGD are introduced, but in the results and discussion are sometimes written in-full).

-Please remove all quotation marks from the quotations in the results section that are two sentences or longer. Quotations should only be indented and italicised when they are two sentences or longer. If they are shorter than two sentences, they are not italicised, and should be integrated into the paragraph (not indented) and DO then have quotation marks.

==============================

Please submit your revised manuscript by Oct 28 2026 11:59PM. If you will need more time than this to complete your revisions, please reply to this message or contact the journal office at plosone@plos.org. When you're ready to submit your revision, log on to https://www.editorialmanager.com/pone/ and select the 'Submissions Needing Revision' folder to locate your manuscript file.

Please include the following items when submitting your revised manuscript:

- A letter that responds to each point raised by the academic editor and reviewer(s). You should upload this letter as a separate file labeled 'Response to Reviewers'.
- A marked-up copy of your manuscript that highlights changes made to the original version. You should upload this as a separate file labeled 'Revised Manuscript with Track Changes'.
- An unmarked version of your revised paper without tracked changes. You should upload this as a separate file labeled 'Manuscript'.

If you would like to make changes to your financial disclosure, please include your updated statement in your cover letter. Guidelines for resubmitting your figure files are available below the reviewer comments at the end of this letter.

If applicable, we recommend that you deposit your laboratory protocols in protocols.io to enhance the reproducibility of your results. Protocols.io assigns your protocol its own identifier (DOI) so that it can be cited independently in the future. For instructions see: https://journals.plos.org/plosone/s/submission-guidelines#loc-laboratory-protocols. Additionally, PLOS One offers an option for publishing peer-reviewed Lab Protocol articles, which describe protocols hosted on protocols.io. Read more information on sharing protocols at https://plos.org/protocols?utm_medium=editorial-email&utm_source=authorletters&utm_campaign=protocols.

As the corresponding author, your ORCID iD is verified in the submission system and will appear in the published article. PLOS supports the use of ORCID, and we encourage all coauthors to register for an ORCID iD and use it as well. Please encourage your coauthors to verify their ORCID iD within the submission system before final acceptance, as unverified ORCID iDs will not appear in the published article. *Only*  the individual author can complete the verification step; PLOS staff *cannot*  verify ORCID iDs on behalf of authors.

We look forward to receiving your revised manuscript.

Kind regards,

Tara Tancred, PhD

Academic Editor

PLOS One

Journal Requirements:

If the reviewer comments include a recommendation to cite specific previously published works, please review and evaluate these publications to determine whether they are relevant and should be cited. There is no requirement to cite these works unless the editor has indicated otherwise.

Please review your reference list to ensure that it is complete and correct. If you have cited papers that have been retracted, please include the rationale for doing so in the manuscript text, or remove these references and replace them with relevant current references. Any changes to the reference list should be mentioned in the rebuttal letter that accompanies your revised manuscript. If you need to cite a retracted article, indicate the article’s retracted status in the References list and also include a citation and full reference for the retraction notice.

[Note: HTML markup is below. Please do not edit.]

Reviewers' comments:

Reviewer's Responses to Questions

**Comments to the Author**

Reviewer #1: All comments have been addressed

Reviewer #2: All comments have been addressed

**********

2. Is the manuscript technically sound, and do the data support the conclusions?

Reviewer #1: Yes

Reviewer #2: (No Response)

**********

3. Has the statistical analysis been performed appropriately and rigorously?

Reviewer #1: Yes

Reviewer #2: (No Response)

**********

4. Have the authors made all data underlying the findings in their manuscript fully available?

The PLOS Data policy

Reviewer #1: Yes

Reviewer #2: Yes

**********

5. Is the manuscript presented in an intelligible fashion and written in standard English?

Reviewer #1: Yes

Reviewer #2: Yes

**********

Reviewer #1: The authors have addressed all previous review comments regarding the methodological description, data analysis, and clarity of result presentation. They have also incorporated the requested critical discussion points. The manuscript is worthy of acceptance.

I noticed a minor error, possibly a typographical one, in Table 4 on page 17. Specifically, the percentages in the “Total” column for the rows “Home delivery” and “Used misoprostol” (69.0% and 92.0%, respectively) appear to be incorrect and should be revised.

Reviewer #2: The authors have addressed previous comments by reviewers; I have just a single comment on the methods section.

The program and intervention are described very comprehensively. The qualitative arm participants are well characterized in the results section. The quantitative component is, however, missing the sample size under the Design and Sampling section.

**********

what does this mean?). If published, this will include your full peer review and any attached files.

If you choose “no”, your identity will remain anonymous but your review may still be made public.

**Do you want your identity to be public for this peer review?**  For information about this choice, including consent withdrawal, please see our Privacy Policy

Reviewer #1: **Yes:**  Yaliso Yaya, PhD, Associate Professor, Western Norway University

Reviewer #2: No

**********

[NOTE: If reviewer comments were submitted as an attachment file, they will be attached to this email and accessible via the submission site. Please log into your account, locate the manuscript record, and check for the action link "View Attachments". If this link does not appear, there are no attachment files.]

To ensure your figures meet our technical requirements, please review our figure guidelines: https://journals.plos.org/plosone/s/figures

You may also use PLOS’s free figure tool, NAAS, to help you prepare publication quality figures: https://journals.plos.org/plosone/s/figures#loc-tools-for-figure-preparation.

NAAS will assess whether your figures meet our technical requirements by comparing each figure against our figure specifications.

# Author comment: Author response to Decision Letter 2

**DOI:** 10.1371/journal.pone.0360788.r004

17 Sep 2026

A point-by-point response to reviewers

PONE-D-25-45507R1: Community-Based Delivery of Life-saving Maternal and Newborn Health Interventions in Vulnerable Ethiopian Communities: Lessons from Implementation Research

Dear Editor,

We, the authors, would like to thank yourself and the reviewers for their valuable comments. Our point-by-point responses to the reviewer and editor are below each comment. We also reviewed to ensure that this manuscript version conforms to the journal style.

Many thanks to the authors for this interesting and well-written manuscript. I have a number of relatively minor revisions suggested below, the most pressing of which are methodological, specifically around providing detail about the analytical processes undertaken. After these are addressed I am happy to see this accepted.

Response: Thank you for the positive and encouraging comments. We have addressed all comments, with particular attention to providing greater detail and clarity on the analytical processes. These revisions have strengthened the methodological transparency and overall quality of the manuscript.

Abstract

-It is more appropriate to simply call the study design a “implementation research, deploying a mixed methods process evaluation” as an “implementation research process evaluation” is not really a study design.

Response: Thank you for this clarification. We have revised the sentence to describe the study as implementation research deploying a mixed-methods process evaluation.

-In your methods description, please add a sentence about your analysis beyond just triangulation.

Response: Revised as suggested to provide additional detail on the analytical approach beyond triangulation.

-Please list the domains in the same order in which they are described in the methods in the main text for better flow.

Response: Revised as suggested to present the domains in the same order as in the Methods section.

Introduction

-Please quantify the burden of PPH deaths and some of the other conditions you noted where possible to highlight the extent of the problem.

Response: Thank you for the valuable feedback. We have quantified the burden of MNH mortality and PPH deaths in the revised manuscript.

-Please provide a bit more detail on the unique features of the Ethiopian geography/health system that make CHWs there so important.

Response: Thank you for these important comments. We have expanded the Introduction section to describe Ethiopia’s geographically dispersed agrarian and pastoralist populations, barriers related to distance, mobility, and access to health facilities, and the structure of the primary healthcare system.

-Please provide a bit more background around the role of CHWs in Ethiopia, as they are considerably more entrenched than in other healthcare systems, and that is an important contextual factor in interpreting your results.

Response: We provided additional background on the HEP/ HEWs and community-based volunteers in linking households with the formal health system. Finally, we clarify that the VHLs build on this existing community health platform rather than representing a stand-alone community health worker program in the Context section

-To really couch this as implementation research, it would be important to note that there are clearly evidence-based practice (e.g. misoprostol for PPH prevention), but there are gaps in their implementation due to the absence of uptake of facility-based care. Hence there is a need to test implementation strategies to support the routine use of misoprostol as needed, and VHL distribution of misoprostol at the household level was one such strategy that was evaluated. Basically, please highlight the evidence-based practices explicitly, the implementation strategy (i.e. VHL-led MNH care including home visits and distribution of misoprostol, IFA, chlorhexidine, and POP), and the importance of their evaluation, specifically via process evaluation.

Response: Thank you for this important comment. We have revised the Introduction to clarify that the intervention package and delivery strategies.

Methods

-Please define the cutoff to be determined as “low” for facility births

Response: We have revised the Methods section to define low facility-delivery coverage as fewer than 30% of expected births occurring in a health facility as a pragmatic program-selection criterion used to identify communities with particularly low uptake of facility-based childbirth and a substantial need for community-based MNH interventions.

-Please explain why RE-AIM was used/how it mapped to the process evaluation domains, which go beyond those in RE-AIM.

Response: We have clarified that RE-AIM served as the overarching framework for structuring the evaluation, while Proctor et al.’s implementation outcomes were used to operationalize the key implementation constructs.

-Program monitoring data is part of the process evaluation. Please in Table 1 remove where you have “process evaluation” and the brackets and just rite “IDIs, FGDs”.

Response: Revised as suggested. Table 1 now lists IDIs and FGDs without the additional “process evaluation” designation.

-The “quantitative component” is not clear. Please provide more detail on exactly what this is (presumably what’s in tables 3 and 4? What is “antepartum”? Why not “antenatal” as is more commonly uses? It’s also not clear why only the first two ANC visits were measured and the first PNC visit? It would be important to see justification for the specific focus on these indicators). Please indicate a sample size (3980?) calculation for this component. Please provide more detail about what specifically what indicators were captured by VHLs.

Response: Thank you for this detailed comment. We have expanded the Methods section to describe the quantitative component as a longitudinal program monitoring cohort of 3,980 eligible pregnant women enrolled during the implementation period and followed by VHLs through three scheduled household contacts: two antenatal contacts during pregnancy and one postpartum contact within the first week after delivery.

We have also detailed the indicators captured by VHLs, including pregnancy identification and enrolment, completion of scheduled contacts, birth-preparedness and facility-delivery counselling, distribution and reported use of IFA, misoprostol, chlorhexidine and POP, place of delivery, referrals, and postpartum follow-up.

-What did the interview and FGD guides include? Please describe these according to each participant type, as presumably they were asked different things. Was any literature used to inform them?

Response: We have expanded the Methods section to describe the qualitative data collection tools and their development. Separate semi-structured guides were used for VHLs, postpartum women, and programme managers/health-system actors, with questions tailored to their respective roles and covering implementation fidelity, reach, safety, acceptability, feasibility, and integration within the primary healthcare system. The guides were informed by the study objectives and evaluation framework, with RE-AIM providing the overarching structure and Proctor’s implementation outcomes informing the key implementation constructs.

-What language were interviews and FGDs carried out in? Was translation necessary (please describe the process of translation if so)?

Response: Interviews and FGDs were conducted in participants’ preferred languages, Amharic and Afaan Oromo, by trained interviewers. Audio recordings were transcribed verbatim and translated into English for analysis.

-How long did interviews and FGDs last?

Response: Interviews lasted a mean of 25 minutes (range: 15–37 minutes), while FGDs lasted a mean of 86 minutes (range: 60–94 minutes).

-There is no detail on analysis provided. Please explain in more detail how qualitative analysis was undertaken, and what quantitative analysis was carried out.

Response: We have revised the Methods section to provide greater detail on both qualitative and quantitative analyses, including the analytical processes used beyond triangulation.

Results

-Please ensure that results are presented in the same order in which the domains appear in Table 1 for better flow.

Response: Revised. The Implementation Fidelity section has been moved to follow Safety, consistent with the order of domains in Table 1.

-Just about the section on “safety”, please provide a representative quotation in support of the paragraph about qualitative findings.

Response: Thank you for this suggestion. We have added a representative quotation to the Safety subsection illustrating participants’ reports of medication-related side effects while indicating that these did not result in treatment discontinuation.

-It’s not quite clear if there were any findings that were unique to a specific participant group, or if there were any divergent views worth flagging? For 90 interviews/FGDs, it’s quite a small amount of qualitative data presented. Are findings as comprehensive as they could be?

Response: We also revisited the qualitative dataset to make participant-group-specific findings and divergent views more explicit. The revised Results distinguish findings reported by VHLs, HEWs, health-centre staff, mothers, and other community/family participants and, where relevant, differences between agrarian and pastoral settings.

-It is unclear what results (if any?) stem from “program monitoring”. Please remove mention of this in the methods and discussion if it was not presented here. If it was, please make this more clear. If program monitoring was the quantitative survey, that needs to be made much more clear.

Response: We have clarified that the programme monitoring data constituted the quantitative longitudinal cohort data collected as part of routine programme implementation. The Methods, Results, and Discussion have been revised accordingly to make this explicit.

Discussion

-It would be important to understand the reach of the evidence-based practices in the absence of VHL delivery. For instance, roughly how widespread is the use of chlorhexidine?

Response: Thank you for this suggestion. We have revised the Discussion to provide greater clarity on the reach and use of the evidence-based practices in the absence of VHL delivery, including chlorhexidine.

-The discussion describes “weak supervision”, but this is not a finding that is emphasised in the results.

Response: We have revised the Discussion to ensure that the interpretation of supervision is consistent with the findings presented in the Results.

-There are more limitations around the approach taken—including qualitative data collection—that can be addressed. For instance, no males/partner perspectives in the pastoralist communities.

-It would also be useful to highlight more where your study team feels future research needs to be directed.

Response: Thank you for these important comments. We have expanded the Limitations section to acknowledge that, although husbands/partners and mothers-in-law were included among qualitative participants, male/partner perspectives were not captured in the pastoralist communities. And further research is also needed to determine whether improved implementation reach translates into sustained use of evidence-based MNH interventions and improved maternal and newborn outcomes.

Typographically

-Throughout, please use “healthcare” instead of “health-care”

-You don’t need to introduce IFA as an acronym in the abstract as it’s not repeated in the abstract.

-Please don’t introduce an acronym unless it is repeated multiple times—otherwise please just write the name in-full (e.g. IPHCSD, HMIS, PHC)

-If you do use an acronym, please use it consistently (e.g. VHL, PPH, and FGD are introduced, but in the results and discussion are sometimes written in-full).

-Please remove all quotation marks from the quotations in the results section that are two sentences or longer. Quotations should only be indented and italicised when they are two sentences or longer. If they are shorter than two sentences, they are not italicised, and should be integrated into the paragraph (not indented) and DO then have quotation marks.

Response: Thank you for identifying these editorial and typographical issues. We have reviewed the manuscript and addressed the comments throughout. Specifically, we have standardized “healthcare”, removed unnecessary acronyms, ensured that retained acronyms are used consistently, and revised the formatting and punctuation of quotations according to the journal’s guidelines.

Review Comments to the Author

I noticed a minor error, possibly a typographical one, in Table 4 on page 17. Specifically, the percentages in the “Total” column for the rows “Home delivery” and “Used misoprostol” (69.0% and 92.0%, respectively) appear to be incorrect and should be revised.

Response: Thank you for identifying this error. The percentages were typographical errors and have been corrected in the revised Table 4.

Attachment Submitted filename: Response to Reviewers.docx

# Editor report: Decision Letter 2

**DOI:** 10.1371/journal.pone.0360788.r005

**Authors:** Tara Tancred

25 Sep 2026

Community-Based Delivery of Lifesaving Maternal and Newborn Health Interventions in Vulnerable Ethiopian Communities: Lessons from Implementation Research

PLOS One

Dear Dr. Tiruneh,

plosone@plos.org. When you're ready to submit your revision, log on to https://www.editorialmanager.com/pone/ and select the 'Submissions Needing Revision' folder to locate your manuscript file.

- A letter that responds to each point raised by the academic editor and reviewer(s). You should upload this letter as a separate file labeled 'Response to Reviewers'.
- A marked-up copy of your manuscript that highlights changes made to the original version. You should upload this as a separate file labeled 'Revised Manuscript with Track Changes'.
- An unmarked version of your revised paper without tracked changes. You should upload this as a separate file labeled 'Manuscript'.

If you would like to make changes to your financial disclosure, please include your updated statement in your cover letter. Guidelines for resubmitting your figure files are available below the reviewer comments at the end of this letter.

If applicable, we recommend that you deposit your laboratory protocols in protocols.io to enhance the reproducibility of your results. Protocols.io assigns your protocol its own identifier (DOI) so that it can be cited independently in the future. For instructions see: https://journals.plos.org/plosone/s/submission-guidelines#loc-laboratory-protocols. Additionally, PLOS One offers an option for publishing peer-reviewed Lab Protocol articles, which describe protocols hosted on protocols.io. Read more information on sharing protocols at https://plos.org/protocols?utm_medium=editorial-email&utm_source=authorletters&utm_campaign=protocols.

As the corresponding author, your ORCID iD is verified in the submission system and will appear in the published article. PLOS supports the use of ORCID, and we encourage all coauthors to register for an ORCID iD and use it as well. Please encourage your coauthors to verify their ORCID iD within the submission system before final acceptance, as unverified ORCID iDs will not appear in the published article. *Only*  the individual author can complete the verification step; PLOS staff *cannot*  verify ORCID iDs on behalf of authors.

We look forward to receiving your revised manuscript.

Kind regards,

Tara Tancred, PhD

Academic Editor

PLOS One

Journal Requirements:

If the reviewer comments include a recommendation to cite specific previously published works, please review and evaluate these publications to determine whether they are relevant and should be cited. There is no requirement to cite these works unless the editor has indicated otherwise.

Please review your reference list to ensure that it is complete and correct. If you have cited papers that have been retracted, please include the rationale for doing so in the manuscript text, or remove these references and replace them with relevant current references. Any changes to the reference list should be mentioned in the rebuttal letter that accompanies your revised manuscript. If you need to cite a retracted article, indicate the article’s retracted status in the References list and also include a citation and full reference for the retraction notice.

Additional Editor Comments:

Many thanks to the authors for this timely revision. Apologies for the back-and-forth, but I am still not quite satisfied with how analysis is described. There are also a few typographical edits outstanding.

-Abstract: In the introduction, it should be Village Health Leader (VHL)-led (not leaders). You write “village health leaders” in full in the methods—please use VHL. Also in the methods, please change the text of the first sentence to “Implementation research deploying a mixed-methods process evaluation across seven health centers and 30 vulnerable communities in the Afar, Somali, and Oromia regions was carried out from December 2023 to July 2025.”

-Please double-check all acronyms throughout for consistency (VHL is introduced, then “village health leader” is still used sometimes (e.g. top of page 8 and throughout)), including removing acronyms that are not repeated.

-Analysis is still inadequately described.

- Please have two separate sections: one for data collection and one for data analysis.
- Coding using ATLAS.ti is not the analytical approach—ATLAS.ti is merely software. Was this a framework analysis? How many people worked on the qualitative analysis? Was any kind of coding framework established, and how? What were the actual steps taken, moving from having a transcript in-hand to moving to the actual sections of the results and the representative quotations. How did this happen? This needs to be very clear. You might consider looking at the COREQ guidelines here that outline some key considerations when reporting on qualitative analysis (as well as reporting data collection, etc.): https://onlinelibrary.wiley.com/pb-assets/assets/17416612/COREQ_Checklist-1556513515737.pdf
- It’s also not clear to the reader how you move from longitudinal data to the results. Did you need to clean the data in any way? How were these analysed? Using what software? What sort of values did you create and how?
- It’s also not quite clear why it is 3980 women. I presume that this simply represents *all*  of the women that VHLs engaged with during the study period? Please make this more explicit in the section on data collection.

[Note: HTML markup is below. Please do not edit.]

[NOTE: If reviewer comments were submitted as an attachment file, they will be attached to this email and accessible via the submission site. Please log into your account, locate the manuscript record, and check for the action link "View Attachments". If this link does not appear, there are no attachment files.]

To ensure your figures meet our technical requirements, please review our figure guidelines: https://journals.plos.org/plosone/s/figures

You may also use PLOS’s free figure tool, NAAS, to help you prepare publication quality figures: https://journals.plos.org/plosone/s/figures#loc-tools-for-figure-preparation.

NAAS will assess whether your figures meet our technical requirements by comparing each figure against our figure specifications.

# Author comment: Author response to Decision Letter 3

**DOI:** 10.1371/journal.pone.0360788.r006

25 Sep 2026

A point-by-point response to reviewers

PONE-D-25-45507R2: Community-Based Delivery of Life-saving Maternal and Newborn Health Interventions in Vulnerable Ethiopian Communities: Lessons from Implementation Research

Additional Editor Comments:

Many thanks to the authors for this timely revision. Apologies for the back-and-forth, but I am still not quite satisfied with how analysis is described. There are also a few typographical edits outstanding.

Response: Thank you for your continued guidance and constructive feedback. We have carefully addressed the remaining comments and made the necessary revisions to improve the data analysis and correct the outstanding typographical issues.

-Abstract: In the introduction, it should be Village Health Leader (VHL)-led (not leaders). You write “village health leaders” in full in the methods—please use VHL. Also in the methods, please change the text of the first sentence to “Implementation research deploying a mixed-methods process evaluation across seven health centers and 30 vulnerable communities in the Afar, Somali, and Oromia regions was carried out from December 2023 to July 2025.”

Response: Thank you for this helpful clarification. We have made the requested revisions in the Abstract and Methods.

-Please double-check all acronyms throughout for consistency (VHL is introduced, then “village health leader” is still used sometimes (e.g. top of page 8 and throughout)), including removing acronyms that are not repeated.

Response: Thank you for highlighting this. We have reviewed the manuscript for consistency in the use of acronyms and terminology. We now use VHL consistently after its first definition and have removed acronyms that were used only once or were not necessary.

-Analysis is still inadequately described.

o Please have two separate sections: one for data collection and one for data analysis.

Response: Thank you for this comment. We have separated the Data Collection and Data Analysis sections. The revised Data Analysis section now provides a detailed description of the analytical approaches used for both the quantitative and qualitative data, see below.

o Coding using ATLAS.ti is not the analytical approach—ATLAS.ti is merely software. Was this a framework analysis? How many people worked on the qualitative analysis? Was any kind of coding framework established, and how? What were the actual steps taken, moving from having a transcript in-hand to moving to the actual sections of the results and the representative quotations. How did this happen? This needs to be very clear. You might consider looking at the COREQ guidelines here that outline some key considerations when reporting on qualitative analysis (as well as reporting data collection, etc.): https://onlinelibrary.wiley.com/pb-assets/assets/17416612/COREQ_Checklist-1556513515737.pdf

Response: Thank you for this important clarification. We have revised the manuscript to clearly distinguish the analytical approach from the software used. The revised manuscript presented as follows,

“Qualitative data were analyzed using deductive and inductive thematic analysis. ATLAS.ti was used for data organization, coding, and management. Two Monitoring, Evaluation, and Research team members from JSI and Amref Health Africa jointly reviewed the transcripts and developed a preliminary coding framework based on the study objectives and data collection guides. We discussed emerging concepts and refined the codes as needed. Related codes were then grouped into broader categories and themes through joint review and discussion. Findings were triangulated across participant groups to identify areas of convergence and divergence and enhance the trustworthiness of the analysis. Illustrative quotations were selected to support the identified themes. The qualitative and quantitative methods were integrated during the interpretation phase to provide a comprehensive description of the study findings and produce the final report.”

o It’s also not clear to the reader how you move from longitudinal data to the results. Did you need to clean the data in any way? How were these analysed? Using what software? What sort of values did you create and how?

Response: Thank you for this comment. We have expanded the description of quantitative data management and analysis. The revised manuscript presented as follows,

“Quantitative data collected through the DHIS2 Tracker were exported for analysis. Longitudinal records were checked for completeness, duplicate records, inconsistent values, and inconsistencies across repeated contacts. Data cleaning, management, and analysis were conducted using Stata version 15. Individual-level records were organized chronologically according to women’s enrolment dates and subsequent VHL contacts. The longitudinal data were then collapsed by visit frequency and administrative region using Stata’s “collapse” command to calculate the frequencies and proportions of the outcome variables.”

o It’s also not quite clear why it is 3980 women. I presume that this simply represents all of the women that VHLs engaged with during the study period? Please make this more explicit in the section on data collection.

Response: Thank you for highlighting this. We have clarified in the Data Collection section that the 3,980 women represent the full number of pregnant women identified and enrolled by VHLs during the study period.

Attachment Submitted filename: Response_to_Reviewers_auresp_3.docx

# Editor report: Decision Letter 3

**DOI:** 10.1371/journal.pone.0360788.r007

**Authors:** Tara Tancred

2 Oct 2026

Community-Based Delivery of Lifesaving Maternal and Newborn Health Interventions in Vulnerable Ethiopian Communities: Lessons from Implementation Research

PONE-D-25-45507R3

Dear Dr. Tiruneh,

We’re pleased to inform you that your manuscript has been judged scientifically suitable for publication and will be formally accepted for publication once it meets all outstanding technical requirements.

Within one week, you’ll receive an e-mail detailing the required amendments. When these have been addressed, you’ll receive a formal acceptance letter and your manuscript will be scheduled for publication.

An invoice will be generated when your article is formally accepted. Please note, if your institution has a publishing partnership with PLOS and your article meets the relevant criteria, all or part of your publication costs will be covered. Please make sure your user information is up-to-date by logging into Editorial Manager at Editorial Manager® and clicking the ‘Update My Information' link at the top of the page. For questions related to billing, please contact billing support.

If your institution or institutions have a press office, please notify them about your upcoming paper to help maximize its impact. If they’ll be preparing press materials, please inform our press team as soon as possible -- no later than 48 hours after receiving the formal acceptance. Your manuscript will remain under strict press embargo until 2 pm Eastern Time on the date of publication. For more information, please contact onepress@plos.org.

Kind regards,

Tara Tancred, PhD

Academic Editor

PLOS One

Additional Editor Comments (optional):

Reviewers' comments:

# Editor report: Acceptance letter

**DOI:** 10.1371/journal.pone.0360788.r008

**Authors:** Tara Tancred

PONE-D-25-45507R3

PLOS One

Dear Dr. Tiruneh,

I'm pleased to inform you that your manuscript has been deemed suitable for publication in PLOS One. Congratulations! Your manuscript is now being handed over to our production team.

At this stage, our production department will prepare your paper for publication. This includes ensuring the following:

* All references, tables, and figures are properly cited

* All relevant supporting information is included in the manuscript submission,

* There are no issues that prevent the paper from being properly typeset

You will receive further instructions from the production team, including instructions on how to review your proof when it is ready. Please keep in mind that we are working through a large volume of accepted articles, so please give us a few days to review your paper and let you know the next and final steps.

Lastly, if your institution or institutions have a press office, please let them know about your upcoming paper now to help maximize its impact. If they'll be preparing press materials, please inform our press team within the next 48 hours. Your manuscript will remain under strict press embargo until 2 pm Eastern Time on the date of publication. For more information, please contact onepress@plos.org.

You will receive an invoice from PLOS for your publication fee after your manuscript has reached the completed accept phase. If you receive an email requesting payment before acceptance or for any other service, this may be a phishing scheme. Learn how to identify phishing emails and protect your accounts at https://explore.plos.org/phishing.

If we can help with anything else, please email us at customercare@plos.org.

Thank you for submitting your work to PLOS One and supporting open access.

Kind regards,

PLOS One Editorial Office Staff

on behalf of

Dr. Tara Tancred

Academic Editor

PLOS One
